# Monazite-Type SmPO_4_ as Potential Nuclear Waste Form: Insights into Radiation Effects from Ion-Beam Irradiation and Atomistic Simulations

**DOI:** 10.3390/ma15103434

**Published:** 2022-05-10

**Authors:** Julia M. Leys, Yaqi Ji, Martina Klinkenberg, Piotr M. Kowalski, Hartmut Schlenz, Stefan Neumeier, Dirk Bosbach, Guido Deissmann

**Affiliations:** 1 Nuclear Waste Management and Reactor Safety, Institute of Energy and Climate Research (IEK-6), Forschungszentrum Jülich GmbH (FZJ), 52425 Jülich, Germany; jiyq790@126.com (Y.J.); m.klinkenberg@fz-juelich.de (M.K.); p.kowalski@fz-juelich.de (P.M.K.); h.schlenz@fz-juelich.de (H.S.); d.bosbach@fz-juelich.de (D.B.); g.deissmann@fz-juelich.de (G.D.); 2 Institute for Applied Materials (IAM), Karlsruhe Institute of Technology (KIT), 76021 Karlsruhe, Germany; 3 Theory and Computation of Energy Materials, Institute of Energy and Climate Research (IEK-13), Forschungszentrum Jülich GmbH (FZJ), 52425 Jülich, Germany; 4 Materials Synthesis and Processing, Institute of Energy and Climate Research (IEK-1), Forschungszentrum Jülich GmbH (FZJ), 52425 Jülich, Germany

**Keywords:** monazite-type SmPO_4_, actinide waste form, radiation damage, ion-beam irradiation, threshold displacement energies

## Abstract

Single-phase monazite-type ceramics are considered as potential host matrices for the conditioning of separated plutonium and minor actinides. Sm-orthophosphates were synthesised and their behaviour under irradiation was investigated with respect to their long-term performance in the repository environment. Sintered SmPO_4_ pellets and thin lamellae were irradiated with 1, 3.5, and 7 MeV Au ions, up to fluences of 5.1 × 10^14^ ions cm^−2^ to simulate ballistic effects of recoiling nuclei resulting from *α*-decay of incorporated actinides. Threshold displacement energies for monazite-type SmPO_4_ subsequently used in SRIM/TRIM simulations were derived from atomistic simulations. Raman spectra obtained from irradiated lamellae revealed vast amorphisation at the highest fluence used, although local annealing effects were observed. The broadened, but still discernible, band of the symmetrical stretching vibration in SmPO_4_ and the negligible increase in P–O bond lengths suggest that amorphisation of monazite is mainly due to a breaking of *Ln*–O bonds. PO_4_ groups show structural disorder in the local environment but seem to behave as tight units. Annealing effects observed during the irradiation experiment and the distinctively lower dose rates incurred in actinide bearing waste forms and potential *α*-radiation-induced annealing effects indicate that SmPO_4_-based waste forms have a high potential for withstanding amorphisation.

## 1. Introduction

Since the 1980s, various single-phase and polyphase ceramic materials have been discussed as potential waste forms for the immobilisation of special nuclear waste streams, such as separated plutonium from civilian sources, excess weapons plutonium, or separated minor actinides (MA: Np, Am, Cm) (e.g., [1,2,3,4,5,6,7,8,9,10]). In this context, monazite-type compounds (i.e., monoclinic lanthanide orthophosphates *Ln*PO_4_) have been subject to particular interest due to their specific physico-chemical properties including high structural flexibility for actinide incorporation, high chemical durability, and high radiation resistance ([11,12,13,14,15,16,17,18,19,20,21,22,23]). Monazite crystallises in the monoclinic system (space group *P* 2_1_/*n*, *Z* = 4) with its structure composed of chains of alternating and edge-sharing *Ln*O_9_ polyhedra and PO_4_ tetrahedra parallel to the *c*-axis; the large and irregular *Ln*O_9_ polyhedra can easily incorporate and accommodate a variety of cations including actinide elements [16,24,25,26].

One important issue for crystalline ceramic waste forms concerns potential property changes in response to radiation-induced phase transformations due to the *α*-decay of the incorporated actinides (e.g., [3,4,27,28]). The *α*-decay of actinide elements (e.g., Th, U, Np, Pu, Am, and Cm) produces *α*-particles (i.e., ^4^He^2+^ nuclei) with energies between 4.5 and 6 MeV and some γ-rays. The recoiling nuclei (energy 70 to 180 keV) contribute mostly to radiation damages in crystalline ceramic materials, producing about 1000 to 2000 atomic displacements due to ballistic effects [4,9,29]. In contrast, despite their significantly higher energy, the *α*-particles induce only about 100 to 200 atomic displacements, mainly due to ionisation effects [29].

Monazites in nature can contain significant amounts of naturally occurring *α*-decaying radionuclides such as thorium (up to 30 wt% ThO_2_) and uranium (up to 16 wt% UO_2_) (cf., [3,27,30,31,32]). In spite of comparatively large *α*-doses received by monazites with high uranium and thorium content over long timescales (i.e., often about 10^19^ to 10^20^ *α* g^−1^; [33,34,35,36]), monazites are usually found in the crystalline state in nature (e.g., [34,36,37,38,39,40,41,42]), independently of their thermal history. Sm-rich monazite with an age of 2.64 Ga containing 16.3 wt% ThO_2_ [43]—*α*-dose approx. 3.5 × 10^20^ *α* g^−1^—showed also no evidence of metamictisation. This lacking evidence for metamictisation and the apparent resistance of monazite in nature to radiation-induced amorphisation were among the key factors leading to consideration of monazite-type ceramics as potential nuclear waste forms [4,27].

Monazite can be amorphised rather easily by ion-beam irradiation (e.g., [41,44,45,46,47,48,49,50]), where dose rates are several orders of magnitude higher than internal *α*-dose rates inflicted on actinide-bearing monazites (e.g., ~1500 to 3000 *α* g^−1^ s^−1^; [33,34,35]). This contrasting behaviour suggests that in geologic environments, the rate of damage recovery exceeds the rate of damage production, consistent with the low critical temperatures *T*_c_ observed for ion-beam-induced amorphisation of monazites (*T*_c_ < 500 K; e.g., [44,45,46,47]), low temperatures (~570 K), and activation energies (<3 eV) required for thermal annealing and recrystallisation (cf., [4,41,47,50,51,52,53]). Moreover, from comparison of the amorphisation behaviour of ion-beam irradiated monazite and self-irradiated ^238^Pu implanted monazite, it was inferred that *α*-radiation-induced annealing might play an important role with respect to the observed crystallinity of actinide-bearing monazites [41,50].

Systematic ion-beam irradiation studies of lanthanide orthophosphates were conducted by Meldrum et al. using 800 keV Kr^2+^ irradiation, following the amorphisation process using selected-area electron diffraction and transmission electron microscopy [46]. The critical temperature *T*_c_ above which no amorphisation can be achieved was found to be lower for *Ln*PO_4_ compounds with monazite structures (*Ln* = La—Gd; *T*_c_: 350 to 490 K) compared to the phosphates of Tb, Tm, Yb, Lu, Sc, and Y that crystallise in the zircon structure (*T*_c_: 480 to 580 K), with a systematic increase with the atomic number of the lanthanide cation [46]. Moreover, monazite- and zircon-structure phosphates reveal distinctively lower critical temperatures than their silicate analogues [27,47]. In general, a higher resilience against radiation-induced phase transitions was observed for *AB*O_4_-type phosphates compared to structurally related silicates [39,47,54].

Due to the size of the Sm^3+^ ion (*r* = 113.2 pm, CN = 9, [55]), SmPO_4_ with a monazite structure can easily incorporate and immobilise relevant radionuclides (e.g., Pu, Am, Cm), which exhibit both slightly larger and slightly smaller cationic radii than Sm^3+^ [56,57]. Therefore, we investigate the potential application of monazite-structured SmPO_4_ as a waste form for specific actinide-containing radioactive waste streams. Here, we focus on the analyses of the effects of internal radiation on the local structure and short-range order in SmPO_4_ monazite, using vibrational spectroscopy to derive conclusions on the potential long-term structural behaviour of an actinide-bearing SmPO_4_ waste form in a geological disposal facility. The damage produced in the monazite structure by *α*-decay of incorporated actinides was simulated by using multiple-energy ion-beam irradiation under controlled experimental conditions. The experimental approach was aligned to previous studies of Picot et al. [48], Nasdala et al. [49], and Deschanels et al. [50], who used triple irradiation with Au ions of 1, 3.5, and 7 MeV energy to induce radiation damage in LaPO_4_ [48,50], La_0.73_Ce_0.27_PO_4_ [48] and CePO_4_ [49], respectively, and used *inter alia* Raman spectroscopy to investigate the effects of ion-beam irradiation on the monazite structure (cf., [42,48,49]).

An important parameter to describe radiation effects in solid materials is the threshold displacement energy, *E*_d_, which constitutes the minimal kinetic energy required to displace an atom from its original position inside the crystal lattice. The *E*_d_ value of a primary knocked-on atom (PKA) is a material property dependent on the nature of the atom and its local structural environment (e.g., [58,59,60,61]). The threshold displacement energies *E*_d_ are decisive parameters when simulating the extent of radiation damage in materials (e.g., calculation of displacements per atom, dpa) with widely used codes such as SRIM/TRIM [62,63] or DART [64,65,66]. However, the displacement energies are generally difficult to obtain by experiments and the values are known mainly for simple oxides but only for relatively few ceramic materials and are unknown for most minerals [29]. According to Meldrum et al., the *E*_d_ values for lanthanide orthophosphates were so far not known [47]. Recently, a couple of atomistic modelling studies reported the derivation of these values for selected monazite-type ceramics [67,68]. Therefore, instead of using default *E*_d_ values implemented in SRIM/TRIM (e.g., [49]) or using those for structurally related silicates such as zircon (e.g., [39,47]), we used atomistic simulations to derive *E*_d_ values for various monazites, such as LaPO_4_ and GdPO_4_ [68], as well as SmPO_4_ [this work].

## 2. Materials and Methods

### 2.1. Sample Preparation and Characterisation

For the production of SmPO_4_ ceramics with monazite structures, a rhabdophane-type precursor phase (SmPO_4_ · *n*H_2_O) was synthesised at ambient temperature via a coprecipitation route, similar to the method described by Boakye et al. [69]. An aqueous Sm-nitrate solution (Alfa Aesar, Thermo Fisher GmbH, Kandel, Germany; >99.9% purity) was mixed with citric acid (CA) as chelating agent in a molar ratio of Sm:CA = 1:2. Subsequently, an excess of phosphoric acid (Merck KGaA, Darmstadt, Germany; EMSURE^®^) (P) was added to this solution (Sm:P = 1:5) and the pH was adjusted to about 10 by adding NH_4_OH (25% *v*/*v*; Merck KGaA, EMSURE^®^) while stirring to obtain quantitative precipitation. After drying at 90 °C, the precipitates were calcined at 550 °C for 3 h. After each thermal treatment step, the materials were ground in an agate mortar. The calcined powders were then pressed into pellets with a 10 mm diameter by uniaxial pressing (60 kN or 765 MPa, resp.) and sintered at 1600 °C for 5 h.

The microstructure and homogeneity of the pellets were investigated by scanning electron microscopy (SEM) and energy dispersive X-ray spectroscopic (EDS) analyses using a Quanta 200F instrument (FEI, Eindhoven, The Netherlands) equipped with an Apollo X silicon drift Detector (EDAX, Weiterstadt, Germany). The measurements were performed in a low-vacuum mode at 60 Pa, using an acceleration voltage of 20 kV and spot size 4. EDS mapping was carried out at a working distance of 10 mm and a magnification of 500×, using a resolution of 512 × 400 pixels with a size of 1.11 × 1.11 µm^2^. X-ray diffraction (XRD) analyses were performed on polished pellets and sintered powders with a D4 ENDEAVOR diffractometer (Bruker AXS GmbH, Karlsruhe, Germany) equipped with a LynxEye detector, using Cu radiation (*λ* Cu K*α*_1_ = 1.5406 Å) at a power setting of 40 kV and 40 mA. XRD patterns were obtained at ambient conditions in the 2 *Θ* range from 10 to 100°/130° (pellet/powder) using a step size of 0.02°/2 *Θ* and a counting time of 0.5/4 s (pellet/powder) per step. EVA software (Bruker AXS GmbH) was used for qualitative phase analysis, as well as for background and *x*-axis correction, and K*α*_2_ stripping for visualisation. For refinement of the Sm-orthophosphate structure, the Rietveld method [70] as implemented in the Topas-Academic software (Coelho Software, Brisbane, Australia; Version V4.1; [71]) was used, applying a fundamental parameter approach for X-ray line profile fitting [72]. In the refinement, sample height/displacement, crystallite size, lattice parameters, and fractional coordinates for all sites, as well as a six-coefficient background polynomial were allowed to vary, using the monazite structure of Ni et al. as a starting model [24].

**Figure 1:**
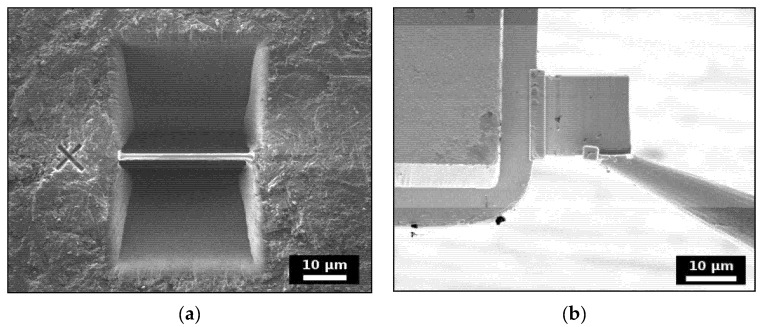
Preparation of FIB-lamellae, (**a**) wedge-shaped trenches dug out with the focused Ga ion-beam on both sides of the sample material covered with a protective Pt layer, (**b**) attachment of lamella to the sample holder (Figures taken from [56]).

SmPO_4_ lamellae for irradiation experiments were prepared from sintered pellets by focused ion-beam (FIB) milling, using a Zeiss NVision 40 Cross Beam workstation (Carl Zeiss AG, Oberkochen, Germany) (cf., Figure 1). The instrument is equipped with a GEMINI high-resolution field emission electron gun and a high performance SIINT zeta FIB column, thus providing for a combination of ion milling and sample characterisation by SEM. Prior to sample preparation, the pellet was coated with a carbon layer to enhance conductivity. A protective layer of around 2 μm thickness of Pt was deposited on the region of interest to protect the sample surface. Afterwards, an approximately 1.5 μm thick slice was cut out of the bulk material using a Ga ion-beam at a power setting of 13 nA and 30 kV, and attached to a Cu sample holder by C deposition. The lamellae were subsequently thinned with a decreasing beam current and finished with a low kV polishing (10 kV/10 pA) to reduce amorphisation and Ga implantation on the sample surface due to the Ga ion-beam. Four lamellae were prepared with a size of about 20 × 15 μm^2^ and a final thickness between 300 and 600 nm. No obvious grain boundaries were observed in the finished lamellae by SEM examination.

Raman spectra were recorded on sintered SmPO_4_ pellets and FIB lamellae at ambient pressure using a Horiba Jobin Yvon LabRam HR (high resolution) Raman spectrometer (HORIBA Jobin Yvon, Villeneuve d’Ascq, France) with a focal length of 80 cm, equipped with a Peltier-cooled charge-coupled device (CCD) detector, using a diffraction grating with 1800 grooves per mm. For excitation, a He-Ne laser (wavelength 632.8 nm) was employed. The spectral resolution, i.e., the instrumental profile function or apparatus function, was evaluated to be 0.8 cm^−1^. Spectra were recorded in the wavenumber range from 100 to 1200 cm^−1^. For each spectrum, an acquisition time of 10 to 25 s (pellet samples) and 60 s (FIB lamellae), respectively, was used, with an average of two accumulated spectra. Multiple measurements were performed across each pellet and lamella, respectively, to consider potential lateral variations. Peak positions were analysed using the Horiba LabSpec software (HORIBA Scientific, Villeneuve d’Ascq, France; version 5.58.25) after fitting the Raman modes using Gaussian–Lorentzian mixed functions. The measured FWHMs of the Raman bands were corrected for the “apparatus function” or “instrumental profile function (IPF)” of the Raman system (cf., [42,73,74,75]), using the empirical formulae of Dijkman and van der Maas [73] and Váczi [76], respectively. Both methods lead to similar true FWHMs; in all cases the corrections amounted to less than 0.2 cm^−1^.

### 2.2. Ion-Beam Irradiation

Three SmPO_4_ pellets and lamellae each were irradiated with multiple-energy Au ions at the *JANNuS* facility at the Commissariat à l’Energie Atomique et aux Energies Alternatives (CEA), Saclay, France, using the *Japet* accelerator. The *Japet* acceleration facility consists of a 6SDII-2 2MV Tandem pelletron-accelerator, equipped with an SNICS II ion source. The samples were irradiated at ambient conditions with equal doses of Au ions with energies of 1 MeV (Au^2+^), 3.5 MeV (Au^3+^), and 7 MeV (Au^5+^), to obtain a rather constant deposited nuclear energy profile throughout the affected depth zone of up to about 1.5 µm (cf., [48,49,50]). The selected fluences per Au ion energy were 2.0 × 10^12^, 6.0 × 10^12^, and 1.7 × 10^14^ ions cm^−2^, respectively, i.e., the total doses of the triple irradiations ranged from F1 = 6.0 × 10^12^, via F2 = 1.8 × 10^13^ to F3 = 5.1 × 10^14^ ions cm^−2^. In order to minimise potential ion channelling in the monazite samples, the ion-beam was directed at an angle of about 15° to the surface of the pellets and lamellae, respectively. The temperature in the sample chamber was kept to about 20 °C. Irradiation-induced changes in the crystallinity of the monazite-structured SmPO_4_ samples were assessed by Raman spectroscopy, comparing the spectra of the irradiated samples to measurements performed on an unirradiated reference pellet and lamella, respectively.

### 2.3. SRIM/TRIM Calculations and Simulation of Threshold Displacement Energies

The penetration depth of the Au ions, the resulting displacements of target atoms, and the distribution of vacancies in the experimentally studied monazite-structured SmPO_4_ were evaluated with the help of the SRIM/TRIM (Stopping and Range of Ions in Matter/Transport of Ions in Matter) software package, using SRIM-2013 (cf., www.srim.org). SRIM/TRIM employs a Monte Carlo (MC) simulation method to address interactions of ions with energies of up to 2 GeV amu^-1^ with matter. It is based on a quantum mechanical treatment of the collisions of incident particles with the atoms in the target material [62,63,77] using the binary collision approximation (BCA) [78]. The results of the SRIM/TRIM simulation results comprise, *inter alia*, the three-dimensional distribution of sputtered ions and vacancies in the target material, as well as the partitioning between nuclear and electronic energy losses, following all target atom cascades in the target material in detail. In the SRIM/TRIM simulations, it is generally assumed that the target is isotropic and amorphous. Moreover, annealing or recrystallisation processes are disregarded. The TRIM simulations of the irradiation experiments were performed for 10,000 incident ions in full cascade mode to obtain sufficient statistical precision. The density of the unirradiated SmPO_4_ was set to 5.3 g cm^−3^ corresponding to the measured density of the sintered SmPO_4_ pellets (i.e., ~94% of the theoretical density of SmPO_4_ with a monazite structure [56]).

The threshold displacement energies *E*_d_ of all three chemical species constituting the investigated material were derived by intensive molecular dynamics simulations using the LAMMPS code [79], adapting the methodologies of Robinson et al. [80] and Ji et al. [68], respectively. In addition to the standard Coulomb interaction term, Buckingham-type interaction potentials between atoms of type *i* and *j*, *Φ_i_*_,*j*_, in the form [81]

(1) $${}_{\Phi_{i,j}}=A\cdot \exp\left(-Br\right)-C/r^{6}$$

were employed in the simulations, with *r* being the distance between interacting atoms. The *A*, *B*, and *C* parameters for the *Ln*–O interactions were fitted to reproduce the *ab initio* data of Blanca-Romero et al. [82]. Regarding the P–O and O–O interactions, the parametrisation of Gale and Henson [83] and Girard et al. [84] were applied, respectively (cf., Table 1); cut-off values of 12 Å were applied for the interatomic distances. The resulting interaction energy is given in eV.

The simulations of the *E*_d_ values were performed using monazite structure supercells containing 1536 atoms, assuming a range of PKA energies for each atom. For each PKA energy, 100 independent simulations were performed with the PKA initial velocity directions distributed randomly or symmetrically on the surface of a sphere with the symmetric arrangement constructed according to the Thomson model (cf., [80]); both simulation methods yielded very similar results. Each simulation was performed for 5 ps allowing for the diminishing of effects of the initial cascade and subsequent system equilibration. An algorithm to analyse displacements and defects according to initial and final atomic positions in the monazite-type lattice was employed to estimate displacement and defect formation probabilities. The simulations were performed assuming *T* = 300 K, controlled by a thermal layer. The threshold displacement energies, *E*_d_, were obtained from the dependence between the initial PKA energy *E* and the displacement probabilities DP(*E*) [80,85], by fitting the equation:

(2) $$\mathrm{DP}\left(E\right)=\left[E^{\alpha }-E_{d}^{\alpha }\right]/\beta ,\;E>E_{d}$$

with *α* and *β* as fitting parameters.

## 3. Results

### 3.1. Material Characterisation

**Figure 2:**
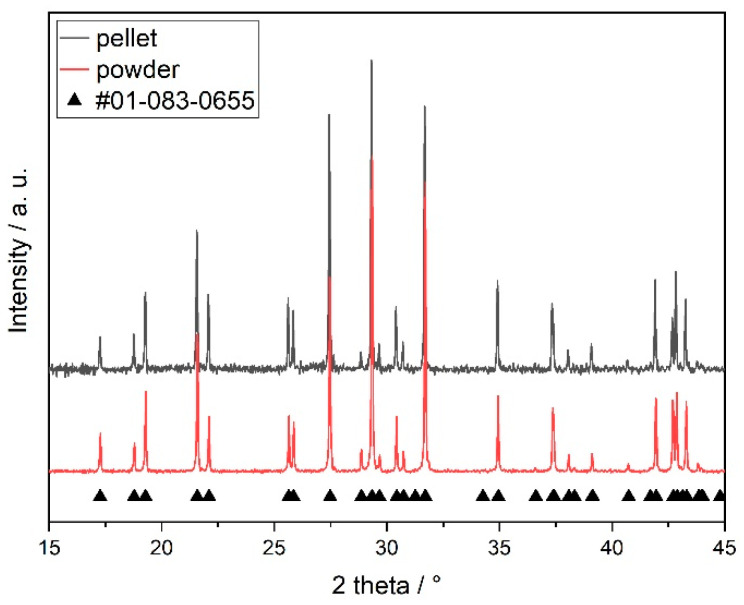
Powder diffraction patterns with normalised intensities of monazite-structured SmPO_4_ powder and pellet samples in comparison to crystal structure data reported by Ni et al. (PDF no. 01-083-0655) [24].

The XRD patterns of sintered SmPO_4_ powder and pellet samples are shown in Figure 2, revealing the typical monoclinic monazite structure. The structural parameters (Table 2) and bond lengths obtained by Rietveld refinement are in good agreement to published structural data, obtained by XRD (e.g., [18,24,86,87,88]) or atomistic simulations [67,82]; however, the DFT data of Rustad [89] systematically overestimate the lattice parameters by a few percent, which is expected from the Perdew–Burke–Ernzerhof (PBE) exchange-correlation functional [90] applied in these studies. No additional phases or impurities were detected by XRD.

**Figure 3:**
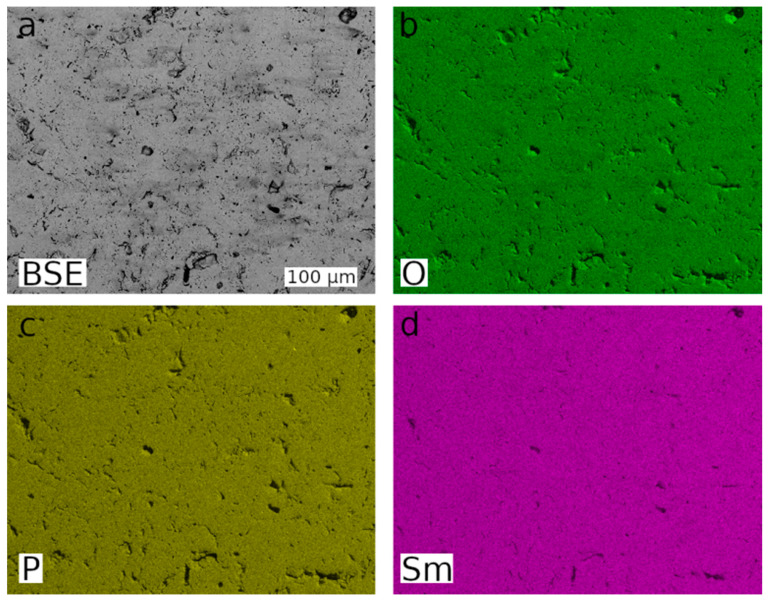
SEM-BSE image (**a**) and results of EDS elemental mapping of the distribution of oxygen (**b**), phosphorous (**c**), and samarium (**d**) performed on a polished SmPO_4_ pellet.

The uniform grey values obtained in SEM pictures of polished pellets taken in back scattered electron (BSE) mode already indicate the chemical homogeneity of the synthesised monazite-structured SmPO_4_ (cf., Figure S1 in the Supplementary Materials). Moreover, the EDS analyses and the elemental mappings performed on polished pellets revealed that, within the error of the applied analytical method and on the scale of the applied methods, the synthesised monazite-structured SmPO_4_ is chemically homogeneous (cf., Figure 3).

**Figure 4:**
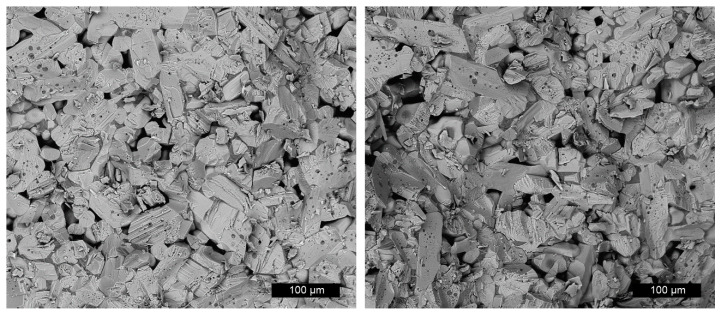
SEM-BSE images taken from the fracture edge of an (intentionally) broken pellet, revealing a dense, nonregular texture with elongated SmPO_4_ grains.

Figure 4 shows SEM-BSE images taken from the fracture edge of an (intentionally) broken sintered SmPO_4_ pellet, revealing a dense, nonregular texture with elongated SmPO_4_ grains without preferential orientation. According to Heuser, the density of the sintered pellets was about 94% of the theoretical density of monazite-structured SmPO_4_ [56]. The SmPO_4_ grains contain partly small (1 to 5 µm), round intergranular pores. Conchoidal fractures as well as fractures along smooth grain boundaries and/or cleavage planes are present. From the SEM pictures, the dimensions of the grains were estimated to be about 100 to 120 µm along the longer axis, and about 10 to 25 µm in cross direction and showed no preferred orientation (cf., also data from optical microscopy in [56]).

### 3.2. Simulation of Threshold Displacement Energies

**Figure 5:**
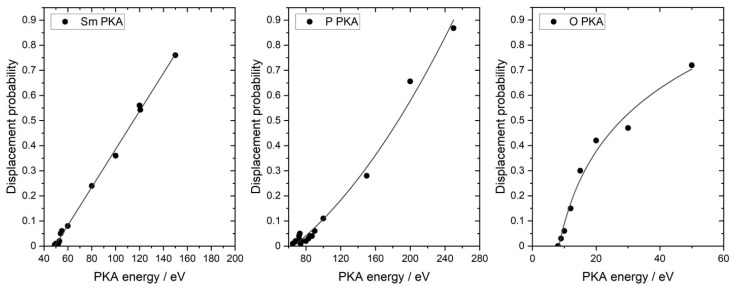
Simulated displacement probabilities of Sm, P, and O PKA in monazite-structured SmPO_4_ as function of PKA energy at *T* = 300 K; the relative error in the data is estimated at ±5%.

The simulated displacement probabilities for the Sm, P, and O atoms in the monazite structure as a function of PKA energy are shown in Figure 5. Each point represents the average value obtained by sampling the 100 directions for a single PKA energy.

The derived *E*_d_ values are 49 eV for Sm, 65 eV for P, and only 8 eV for O, indicating that it is easiest to form a defect at the oxygen site and hardest to displace the P atoms in the phosphate tetrahedra in the SmPO_4_ lattice. Due to the high P–O bond strength compared to the Sm–O bond, the P atoms are “protected” against displacement within the PO_4_ tetrahedra. Moreover, the ionic radius of P (17 pm) is significantly smaller compared to O (138 pm) and Sm (113.2 pm) in the monazite structure [55]; hence, the probability of striking a P atom in a displacement cascade is lower. Similar trends with respect to the *E*_d_ values have been obtained previously [68] in atomistic simulation studies addressing monazite structures containing La and/or Gd (cf., Section 4.1). The *E*_d_ values for oxygen are practically identical (~8 eV); however, with respect to the *E*_d_ values of the *A* and *B* cations, the simulation results show lower values for SmPO_4_ compared to the terminal monazite-type phases within the monazite-type series (*Ln*PO_4_, *Ln* = La—Gd), in particular for P. A similar observation was made for monazite-structured (La,Eu)PO_4_, where a minimum of *E*_d_ values for the *Ln*-cations within this monazite-type solid solution series was calculated for an Eu mole fraction of 0.2 [91]. The authors interpreted the observed minimum as a consequence of two opposing effects: (i) the decrease of the unit cell volume with increasing Eu content, impeding the displacement of the *Ln*-cations, and (ii) the weakening of the *Ln*–O bond with an increasing *Ln* atomic number, facilitating the displacement of the *Ln*-cations [91]. These opposing effects can probably also serve as an explanation for the lower *E*_d_ values for Sm and P in SmPO_4_ compared to the values derived for the *A* and *B* cations in LaPO_4_ and GdPO_4_.

### 3.3. SRIM/TRIM Simulations

**Figure 6:**
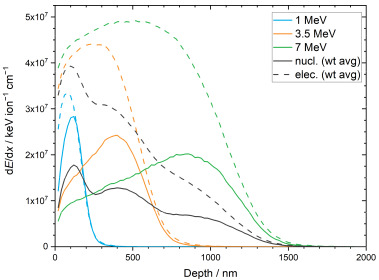
Simulated energy deposition in monazite-structured SmPO_4_ by nuclear (continuous lines) and electronic (dashed lines) stopping processes due to irradiation with Au ions of different energies (individual ions and weighted average for triple irradiation).

SRIM/TRIM simulations of the triple irradiation of monazite-structured SmPO_4_ with Au ions were performed in support of the interpretation of the results of the ion-beam irradiation experiments. Figure 6 shows the simulated energy deposition by nuclear stopping processes (i.e., elastic collisions between the Au projectile ions or recoiling nuclei with atoms in the monazite structure) and electronic stopping processes (i.e., inelastic collision between bound electrons in the monazite structure and the moving Au ions or recoiling nuclei) for the individual irradiations as a function of sample depth. The average energy deposition and doses are summarised in Table 3. During triple irradiation, about 69% of the energy of the incoming Au ions is deposited by electronic stopping processes; the energy deposition by nuclear stopping processes amounts to ~31%.

**Figure 7:**
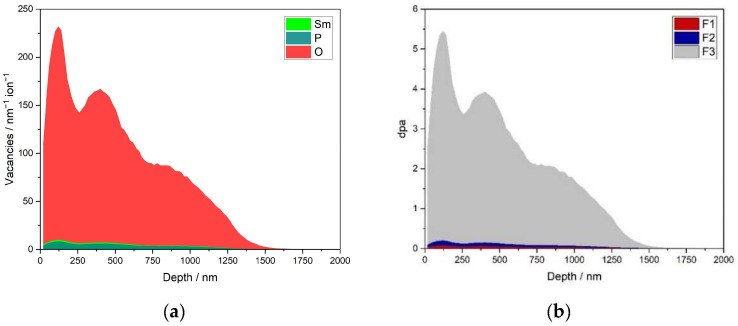
Simulated vacancy distribution in monazite-structured SmPO_4_ after triple irradiation with Au ions (1, 3.5, and 7 MeV) (**a**), and calculated dpa dose vs. depth for the various fluences used in the irradiation experiments (**b**).

The simulated vacancy distribution in the monazite-structured SmPO_4_ due to triple irradiation with Au ions (1, 3.5, and 7 MeV) and the calculated dpa dose for the fluences used in the irradiation experiments are shown in Figure 7 as a function of sample depth. The results of the SRIM/TRIM simulations are summarised in Table 4. According to the simulations, the maximum in the number of vacancies is located at a distance of about 120 nm from the sample surface; the irradiation affected zone extends to a depth of about 1640 nm in bulk SmPO_4_ with a monazite structure. As expected from the displacement threshold energies, mainly O vacancies are created, whereas the number of Sm and P vacancies are relatively low. The simulated depth profiles indicate that the thickness of the irradiated lamellae (300 to 600 nm) is significantly smaller than the depth range affected by irradiation-induced damage in SmPO_4_, suggesting that the lamellae could be completely amorphised in the ion-beam irradiation experiments at sufficiently high fluence, as long as the temperature during the experiment is below *T*_c_. The simulated profiles indicate that, due to the application of triple irradiations with different energies at equal fluences, a damage gradient is expected to develop in the irradiated SmPO_4_ material. However, the damage predicted in the irradiated FIB lamellae (300 to 600 nm thickness) is rather similar (on average ~180 to 190 vacancies ion^−1^ nm^−1^) with a relative standard deviation of approx. 20%.

The simulated depth of the irradiation-affected layer on the surface of bulk SmPO_4_ (e.g., when irradiating pellet samples or (larger) single crystals) agrees well with experimental results obtained on Au ion irradiated La-monazite pellets by Deschanels et al., using a triple irradiation scheme similar to the one in this study [50].

Applying BF-TEM and selected area diffraction, these authors found amorphisation in La-monazite up to 1.6 µm after irradiation at a fluence of 7.2 × 10^14^ ions cm^−2^.

### 3.4. Ion-Beam Irradiation of SmPO_4_

Raman microspectroscopic analyses were performed on pristine monazite-structured SmPO_4_ samples and SmPO_4_ pellets and lamellae after multienergy Au ion irradiation. In Raman spectroscopy, increasing short-range disorder is commonly characterised by band broadening, using the FWHMs as a measure for disorder in crystal structures (e.g., [36,42,49,75,92]). The *ν*_1_ symmetric stretching band of the PO_4_ tetrahedra (~980 cm^−1^) was used for assessing the short-range order of the monazite-structured SmPO_4_, due to its high intensity even in (partially) radiation-damaged samples, and the lack of significant orientation dependence of the Raman shift and the bandwidth (cf., [36]). The sensitivity of the *ν*_1_ vibration of monazite-type phases to structural disorder has already been demonstrated, for example, by Seydoux-Guillaume et al. [33], Nasdala et al. [49], Ruschel et al. [36], and Nasdala et al. [42]. Raman stretching modes are specifically sensitive to local disorder of nearest neighbour structural atoms, particularly atoms from other sublattices [92]. Table 5 provides the *ν*_1_ vibration band positions and FWHMs of unirradiated and irradiated SmPO_4_ samples.

**Figure 8:**
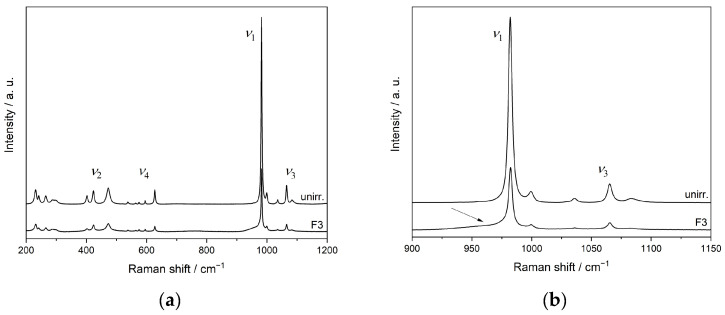
Raman spectra of a pristine SmPO_4_ pellet and the pellet irradiated with the highest fluence (F3 = 5.1 × 10^14^ ions cm^−2^) ((**a**) total spectrum, (**b**) region of stretching vibrations *ν*_1_ and *ν*_3_); the arrow indicates a shoulder developing at a wavenumber around 960 cm^−1^ (Figures reproduced from [56]).

Figure 8 shows Raman spectra of pristine and Au-irradiated SmPO_4_ pellets. The position of the *ν*_1_ band in the unirradiated material at 982.0 cm^−1^ is in agreement with the data from Begun et al. [93] and Ruschel et al. [36], obtained on SmPO_4_ single crystals (*ν*_1_(PO_4_): 982 cm^−1^ and 981.3 cm^−1^, respectively). Silva et al. [94] determined a slightly higher value for the position of the *ν*_1_ band of SmPO_4_ in monazite structure (983 cm^−1^). However, the FWHM of the *ν*_1_ band (averaged over a number of measurements performed on three unpolished pellets) of the unirradiated material was found to be somewhat higher than the one from Ruschel et al. [36] (FWHM 2.6 cm^−1^), potentially due to the polycrystalline nature of the material used in the investigations of this study.

The spectra of the pellets irradiated at fluences F1 and F2 are rather similar to the unirradiated crystalline reference sample; however, a decrease in intensities was observed, and, therefore, not included in Figure 8. Only at the highest fluence (5.1 × 10^14^ ions cm^−2^, average dpa = 2.13), a slight change around the band of the *ν*_1_ vibration of the phosphate tetrahedron was observed, leading to the development of a shoulder with higher intensity at lower wavenumbers, indicating an amorphisation at the pellet surface (cf., Figure 8, right). Based on the SRIM/TRIM calculations, the irradiation-affected zone extends to about 1600 nm below the pellet surface. In contrast, the depth of the volume analysed by Raman spectrometry is distinctively larger (cf., [49]). Moreover, the Raman scattering from the crystalline bulk material beneath the irradiation-affected zone is more intense than from amorphous material (cf., [42,49]). Thus, the Raman spectra obtained from irradiated pellet samples show mainly contributions from the underlying crystalline material, obscuring the observation of irradiation-induced structural changes close to the sample surface, despite the calculated average defect densities of up to about dpa = 2.1 in the irradiation affected surface layer.

**Figure 9:**
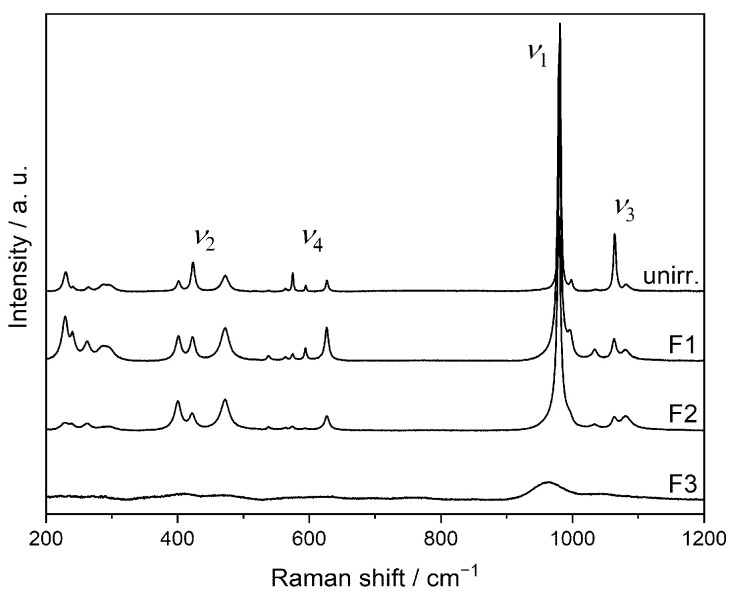
Raman spectra of SmPO_4_ FIB lamellae after triple irradiation with multienergy Au ions at different fluences compared to unirradiated monazite-structured SmPO_4_ (Fig. reproduced from [56]).

In Figure 9, the representative Raman spectra of nonirradiated and Au ion irradiated FIB lamellae are shown. The thickness of the lamellae is significantly smaller than the calculated penetration depth of the Au ions and the extent of the irradiation-induced damaged zone; thus, the ion irradiation affects the complete lamellae, inflicting calculated average damages of 0.05, 0.15, and 3.81 dpa in the monazite-structured SmPO_4_ materials, respectively. The Raman spectrum of the nonirradiated lamella is rather similar to the one of the pristine SmPO_4_ pellet. However, a slight shift in the position of the *ν*_1_ band to lower wavenumbers (980.9 cm^−1^) was observed, compared to the unirradiated pellet. This shift, similar to the one observed for monazite-(Ce) lamellae and single crystals by Nasdala et al. [49], as well as the higher FWHM compared to data from SmPO_4_ single crystals [36] can probably be attributed to contributions of surface effects and/or local strain in the foils from sample preparation (cf., [42,49,95]). With increasing doses, a broadening of the Raman bands accompanied by frequency shifts towards lower wavenumbers and decreasing intensities are observed that are typical for radiation-damaged minerals/materials (incl. *Ln*-orthophosphates), indicating a decrease in crystallinity (e.g., [33,42,49,96]). The thickness of the investigated FIB lamellae was in the order of the wavelength of the laser used for the Raman spectroscopy, which can result in the broadening of Raman bands due to surface strain or other effects (cf., [42,49,95]). Here, these effects seem to be small compared to the irradiation-induced changes provoked in the differently irradiated lamellae, thus not impeding the general conclusions of this work.

**Figure 10:**
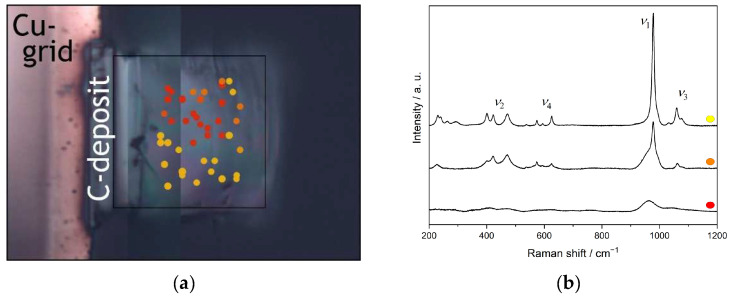
Raman measurement points on SmPO_4_ FIB lamellae (**a**) and respective spectra (**b**) indicating variations in crystallinity in the sample after triple irradiation with multi-energy Au ions at the max. total fluence (F3) of 5.1 × 10^14^ ions cm^−2^; the colour coding of the measurement points (**a**) refers to areas of different crystallinity as indicated in the Raman spectra (**b**) (Figures modified (**a**) and reproduced (**b**) from [56]).

At the highest fluence F3, instead of a peak of the symmetrical stretching vibration *ν*_1_, only a broad bump at ~962 cm^−1^ is visible in an otherwise nearly featureless spectrum, indicating amorphisation including distortion of the phosphate tetrahedra. Nasdala et al. [42] assigned a low-intensity broad bump centred near 950 cm^−1^ in the Raman spectrum of irradiated monazite-(Ce) (i.e., red shift of ~20 cm^−1^ compared to the unirradiated material) to the Raman signal of amorphous CePO_4_. However, Raman spectra obtained at different measuring points on this lamella reveal an inhomogeneous distribution of areas of varying crystallinity in the sample, ranging from nearly crystalline, via intermediate to completely amorphised areas (Figure 10a). Exemplarily, the Raman spectra from areas of different crystallinity in this sample are depicted in Figure 10b. Due to the small facial area of the lamellae (20 × 15 μm^2^) compared to the beam size (2 × 2 cm^2^), a nonuniform irradiation of the lamellae is very unlikely to be the cause for the varying crystallinity. The temperature set in the irradiation chamber during the experiment (~293 K) was well below the critical temperature for SmPO_4_ with a monazite-structure of 472 K as determined by Meldrum et al. [46]. However, slight heating of the samples themselves during ion-beam irradiation, thus facilitating annealing effects, cannot be ruled out. Thus, the varying degree of crystallinity in this sample is assigned to local recrystallisation/annealing effects occurring during or shortly after the irradiation. Repeated Raman measurements performed after a 6-month interval revealed no additional recovery of amorphised areas, indicating that the energy from the laser-beam of the Raman spectrometer does not contribute significantly to further sample annealing. In contrast, the two lamellae irradiated at lower fluences each revealed rather similar Raman spectra at different measurement positions.

**Figure 11:**
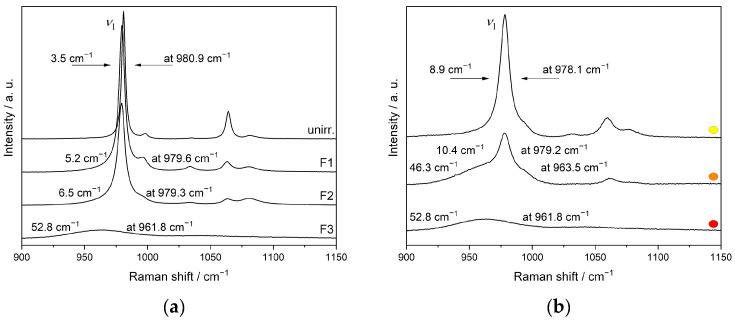
Raman spectra depicting the region of the stretching vibrations in unirradiated and Au ion irradiated FIB lamellae prepared from monazite-structured SmPO_4_, showing the broadening and shift of the symmetrical stretching vibration *ν*_1_ with increasing fluence from F1 to F3 ((**a**); cf., Figure 9), and for variable crystalline regions in the lamella irradiated at fluence F3 ((**b**), cf., Figure 10) (Figures reproduced from [56]).

Figure 11 depicts the region of the stretching vibrations in the Raman spectra of the monazite-structured SmPO_4_ lamellae irradiated at different fluences, including the spectra obtained from different crystalline areas in the material irradiated at the maximum fluence used in this study. Here, the broadening of the Raman bands (increase in FWHM) and the concomitant shift to lower wavenumbers with increasing dose and amorphisation are clearly discernible for the band of the symmetrical stretching vibration *ν*_1_. Compared to the sample irradiated at fluence F2 to an average dpa = 0.15 (FWHM 6.5 cm^−1^), the more crystalline areas in the sample irradiated with the highest dose (5.1 × 10^14^ ions cm^−2^, average dpa = 3.81) also show a higher level of irradiation-induced damage in the crystal lattice, as indicated by the higher FWHM (8.9 cm^−1^) and stronger red-shift. The shift of the Raman bands to lower wavenumbers can be attributed to a decreasing short-range order and the radiation-induced stretching of the P–O bonds and changes in the local environment of the phosphate tetrahedra (e.g., distortion and tilting), as well as the presence of amorphous clusters causing strain due to volume expansion (cf., [49,50]).

The radiation-induced changes in the length of the P–O bonds were estimated from the shift of the *ν*_1_ band using the approach of Popovic et al. [97]. The red-shift of the *ν*_1_ vibration band indicates only a small P–O bond length increase from 155.1 to 155.6 pm as a consequence of the Au ion irradiation even in the sample irradiated at the highest fluence. This small increase in the P–O bond length indicates that these bonds are not broken and the phosphate tetrahedra behave either as tight units under irradiation [48] or can recombine rather quickly after receiving irradiation damage. This supports the assumption that amorphisation of monazite occurred mainly due to increasing (short-range) structural disorder in the local environment of the phosphate groups (cf., [36,49]) and breaking of *Ln*–O bonds. Since all O atoms belong to SmO_9_ polyhedra, as well as to PO_4_ tetrahedra, both polyhedra are destroyed when an O atom is displaced. However, the resulting P^V^O_3_ units are highly unstable and directly capture nearby O atoms. Therefore, tilted/distorted and recombined PO_4_ tetrahedra are observed after irradiation, although P–O bond lengths, O–P–O angles, and the sites of P atoms have probably changed. Moreover, the vibrations of the P–O bonds are eased due to the destruction of the SmO_9_ polyhedra, which contributes to the shift to lower vibration frequencies.

## 4. Discussion

### 4.1. Radiation Damage in Monazite-Type LnPO_4_

The comparison of the results of different studies on ion-beam irradiation of natural and synthetic *Ln*-orthophosphates with a monazite structure, for example, with respect to the dpa and fluences required for amorphisation, is not straightforward, since often monazite-type samples of different compositions were investigated using varying irradiation conditions. Moreover, different approaches regarding the selection of displacement threshold energies used for the calculation of dpa were applied. In this study, *E*_d_ values derived from atomistic simulation were used. In Table 6, the *E*_d_ values of the current study are compared to those used in previous studies of radiation effects in various monazite-type phosphates, where, in many cases, rather uniform *E*_d_ values for all atoms in the monazite structure were used (e.g., [44,45,46,49,50]). These are generally lower for the *Ln* and P atoms and higher for O atoms compared to the *E*_d_ values obtained by molecular modelling. Meldrum et al. [39,47] used *E*_d_ values for zircon (ZrSiO_4_) obtained by molecular dynamic simulations [4,98] for monazite and synthetic LaPO_4_, respectively. Interestingly, for the zircon *AB*O_4_ structure, the lowest *E*_d_ value has been assigned to the atom on the *B* position, whereas, for oxygen, a relatively high *E*_d_ value was obtained (47 eV). On the other hand, molecular dynamics simulations of displacement energies in zircon by Park et al. [99] revealed a contrasting trend (*E*_d_ 89 eV for Zr, 48 eV for Si, and 28 eV for O). This can probably be attributed to different force-field parameterisation used in these studies.

By using triple irradiation with multienergy Au ions (1, 3.5, and 7 MeV) in this study, first indications of structural modifications in monazite-structured SmPO_4_ were already observed in the Raman spectra at an average dpa of 0.05 to 0.15; vast amorphisation was achieved at a fluence of 5.1 × 10^14^ ions cm^−2^ and an average dpa of 3.81, notwithstanding that local annealing occurred. This is in the order of magnitude of the critical amorphisation dose (fluence 1.21 × 10^14^ ions cm^−2^, reported dpa = 0.38) reported by Meldrum et al. to cause amorphisation of monazite-structured SmPO_4_ using irradiation with 800 keV Kr^2+^ ions [46]. In these experiments, amorphisation of various monazites (*Ln*: La—Gd) was generally achieved at a fluence of about 1.2 × 10^14^ ions cm^−2^ (reported dpa 0.35 to 0.39 [46]). Picot et al. concluded from grazing incidence XRD patterns a practically complete amorphisation of LaPO_4_ at a fluence of 7.2 × 10^14^ ions cm^−2^ (reported dpa = 2.11) when using the same triple irradiation scheme with Au ions as in the present study [48]. Similarly, Nasdala et al. [49] and, more recently, Deschanels et al. [50] investigated multienergy (1, 3.5, 7 MeV) Au ion-irradiation-induced phase transformations in Ce-monazite and La-monazite, respectively. Similar to the here presented results, Nasdala et al. achieved amorphisation of CePO_4_ at a fluence of 5.1 × 10^14^ ions cm^−2^ (reported dpa = 1.0) [49]. Recently, Nasdala et al. observed complete amorphisation in foils of synthetic CePO_4_ and monazite-(Ce) from nature in triple irradiation experiments (1, 4, 10 MeV Au-ions) already at a fluence between 4.5 × 10^13^ and of 1.2 × 10^14^ ions cm^−2^ (corresponding to reported dpa values of 0.125 and 0.353, respectively) [42]. The lower amorphisation fluences and higher damage compared to the previous study were attributed to the fact that irradiation was performed at a low temperature (liquid N_2_ cooling), leading to a significantly reduced fraction of immediate defect recombinations. Using TEM investigations, Deschanels et al. observed complete amorphisation of LaPO_4_ up to a depth of 1.6 µm from the surface at a fluence of 7.2 × 10^14^ ions cm^−2^ (reported dpa = 2.11); amorphisation was seen to occur for fluences exceeding 9.6 × 10^13^ ions cm^−2^ (reported dpa = 0.28), while, at a lower fluence of 1.3 × 10^13^ ions cm^−2^ (dpa = 0.05), the deposited energy was not sufficient to lead to discernible amorphisation; however, it resulted in a strained lattice up to 1.2 µm depth [50]. Similarly, Seydoux-Guillaume et al. achieved complete amorphisation of Au-irradiated LaPO_4_ foils with a monazite structure at 2 × 10^14^ ions cm^−2^ (reported dpa 1.13); amorphisation was found to be almost complete already at 1 × 10^14^ ions cm^−2^ (reported dpa 0.56), with only few phantoms of grain boundaries remaining [41]. However, it has to be noted that the degree of radiation damage detected in the materials can differ substantially depending on the analytical techniques employed (e.g., TEM, Raman spectroscopy, photoluminescence or XRD), and thus amorphisation may be detected at different fluences with different methods (cf., [42]).

Thus, the studies using similar irradiation conditions agree rather well on the fluence required for amorphisation of chemically different monazite-structured materials, although the reported dpa values vary distinctively due to different *E*_d_ values used in SRIM/TRIM simulations. Moreover, it often remains unclear whether the reported dpa values refer to maximum or average values in the irradiation-damaged zones. In contrast, in recent ion-beam irradiation studies on monazite-type La_0.2_Gd_0.8_PO_4_ using swift heavy ions (100 MeV Au^9+^), significant amorphisation effects were already observed by XRD at fluences of 10^13^ ions cm^−2^ [68]; full amorphisation of the samples up to a depth of 9 µm was attained at fluences higher than 10^13^ ions cm^−2^. The comparatively low fluences required for amorphisation of the monazite structure by swift heavy ions were attributed to the thermal spike induced by electronic stopping effects [68], in analogy to results of swift heavy ion irradiation experiments using pyrochlore-type materials [100]. Using the *E*_d_ values for LaPO_4_ and GdPO_4_ derived by Ji et al. ([68]; cf. Table 3), it was estimated from SRIM/TRIM simulations in this study that about 95% of the energy of the 100 MeV Au ions was deposited by electronic processes in these irradiation experiments.

Compared to studies using ion-beam irradiation, investigations on the effects of self-irradiation of *Ln*-monazites due to internal alpha-decay of incorporated (short-lived) actinides such as ^238^Pu (half-life 87.7 years, ENDF/B-VIII.0, Brown et al. 2018) are rather scarce, and addressed mainly La-based monazites [50,101]. Burakov et al. reported that ^238^Pu-doped La-monazite (8.1 wt% ^238^Pu) remained crystalline up to a cumulative dose of 2.5 × 10^18^ *α* g^−1^ (i.e., ~0.26 dpa) without any swelling or crack formation [101]. Luo and Liu reported that LuPO_4_ doped with ^244^Cm (half-life 18.11 years, ENDF/B-VIII.0, [102]) remained crystalline even after having received a dose of 5 × 10^19^ *α* g^−1^ [103]. Investigations of Deschanels et al. revealed that, in contrast to externally Au ion irradiated monazite-type materials, ^238^Pu-doped monazite-type samples (La_0.73_Pu_0.09_Ca_0.09_Th_0.09_PO_4_) remained crystalline up to a cumulative dose of 7.5 × 10^18^ *α* g^−1^ (0.8 dpa) accompanied by a low swelling of close to 1% [50]. This contrasting behaviour was attributed to *α*-radiation-induced annealing in actinide-bearing monazite-structured samples and the significantly higher dose rates inflicted in ion-beam irradiation experiments. Recently, Seydoux-Guillaume et al. inferred an *α*-healing-induced defect recovery mechanism from experiments using sequential and simultaneous irradiation of synthetic monazite-type LaPO_4_ with Au and He ions [41]. In contrast, Nasdala et al. observed that He irradiation induced radiation damage in crystalline CePO_4_ [104]. According to Nasdala et al., more research is required to determine in which materials and under which conditions *α*-radiation leads to structural damage and/or can anneal already existing radiation damage, in order to evaluate the significance of these effects for the long-term structural properties of monazite-type materials [42].

Generally, the question about the behaviour of PO_4_ units under irradiation has been discussed controversially. While Picot et al. [48] proposed PO_4_-tetrahedra as particularly irradiation-resistant structural elements, Nasdala et al. [42,49] and Deschanels et al. [50] reported the opposite. These contrasting observations might partially be explained by the competition between the accumulation of irradiation-induced structural damage on the one hand, and immediate and/or fast postirradiation annealing on the other hand, which is rather effective in monazite-structured *Ln*PO_4_ even at low temperatures. Moreover, if ion-beam experiments are performed without sample cooling, the temperature rise in the samples can result in a distinct increase in immediate defect recombinations (cf., [42]).

### 4.2. Implications for SmPO_4_ as Waste Form for Actinides

Application of ceramic waste forms for the immobilisation of actinides can be envisaged in particular for the disposal of surplus Pu from civilian or, if applicable, military sources (e.g., [5,7,10,105]). The investigations of Arinicheva et al. on monazite-type La_1-*x*_Pu*_x_*PO_4_ showed that up to 15 mol% Pu can be incorporated into the monazite structure as Pu^3+^ [106], i.e., no coupled substitution for charge compensation would be required to immobilise separated Pu, which is usually stored as PuO_2_, in such waste matrices. In Pu waste forms, the various alpha decay events in the decay chains of the different Pu isotopes contribute to a variable extent to the total radiation damages arising with time. Within a period of 10^6^ years, in the ^239^Pu and ^242^Pu decay chains, only the decay events ^239^Pu to ^235^U and ^242^Pu to ^238^U, respectively, play a role with respect to potential radiation damages, whereas in the ^238^Pu and ^241^Pu series, practically the complete decay chain contributes to damage events in the longer term, i.e., at times exceeding 10^5^ years (cf., Tables S1 and S2 in the Supplementary Materials). The isotopic composition of civilian plutonium from the reprocessing of spent nuclear fuels depends on various factors, including the type of nuclear reactor and its neutron spectrum, the mode of reactor operation, the initial level of uranium enrichment in the fuel, and the burn-up of the fuel at the time of discharge (cf., Table S3 in the Supplementary Materials). The majority of the Pu consists of the isotopes ^239^Pu and ^240^Pu, with the proportion of the heavier isotopes (^241^Pu and ^242^Pu) increasing with increasing fuel burn-up (e.g., [107]). Depending on the source of Pu, a waste form containing 10 wt% Pu will incur about 1 to 4·10^20^ alpha decay events in 10^6^ years, with average dose rates decreasing from about 0.5 to 1 × 10^9^ *α* g^−1^ s^−1^ in the first thousand years to about 10^7^ *α* g^−1^ s^−1^ over a period of one million years (cf., Table S4).

Inferring potential radiation damages and amorphisation effects occurring in ceramic waste forms in the long-term from irradiation experiments with heavy ions, doping with short-lived alpha emitters, or natural analogues requires the consideration of differences regarding the total received doses (e.g., in dpa), dose rates, and the energy deposition (i.e., electronic vs. nuclear stopping) in the experiments and the waste forms. In Table 7, the calculated alpha doses and alpha dose rates incurred in Pu waste forms (10 wt% Pu loading) in one million years are compared to those received by nonmetamict monazites from nature and alpha-doped *Ln*-phosphates with a monazite structure, respectively. Here, the mode of energy deposition from alpha particles and recoiling nuclei is rather similar in all materials, with electronic energy losses distinctively exceeding energy losses by nuclear processes (cf., [50]). It has to be noted that the total doses received by the waste forms within one million years are in the same order of magnitude as in naturally occurring nonmetamict monazites, though the dose rates are several orders of magnitude higher for the former. However, the total doses in alpha-doped materials within the experimental time frames are about one to two orders of magnitude lower than ones in a waste form integrated over one million years; however, the dose rates in the experiments are distinctively higher due to the usage of short-lived isotopes.

The fast annealing of damages in monazites in nature and alpha-doped synthetic *Ln*PO_4_, despite high alpha doses and/or dose rates, is probably due to the low critical temperature of monazite and alpha-assisted annealing effects [41,108] in consequence of electronic energy losses of alpha particles. Based on SRIM/TRIM simulations of the alpha decays in the Pu decay chains in SmPO_4_, the alpha energy is dissipated to >99.6% by electronic stopping. A similar behaviour can also be expected in Pu waste forms based on monazite-structured *Ln*PO_4_, such as SmPO_4_. In addition to alpha-assisted annealing, Mir and Peuget suggested that additionally, electronic energy losses of recoiling nuclei in waste forms will potentially lead to partial defect healing in the long term (according to SRIM/TRIM simulations, in SmPO_4_, the main energy loss (60 to 65%) of the recoiling nuclei in the Pu decay chains is due to nuclear stopping) [109].

In the present irradiation experiments with SmPO_4_ lamellae, a distinct amorphisation was only observed at the highest fluence, leading to an average dose of 3.8 dpa (dose rate: 2.2 × 10^−4^ dpa s^−1^), though annealing effects were already observed in the course of the experiment. In a real Pu waste form, a time frame of several hundred years would be required to reach a similar damage level, disregarding annealing processes (cf., Tables S2 and S4 in the Supplementary Materials). Considering the radiation stability of alpha-doped synthetic *Ln*PO_4_, the distinctly higher proportion of electronic energy losses in a Pu waste form and the lower dose rates suggest that waste forms consisting of monazite-structured SmPO_4_ will not undergo a macroscopic crystalline to amorphous phase transition in the repository environment. However, it should be noted that in aged (i.e., >10 years) synthetic *Ln*PO_4_ (*Ln* = La, Eu) doped with significant amounts of ^238^Pu, despite preservation of crystallinity, a loss of mechanical integrity and formation of secondary phases (Pu-containing rhabdophane) after storage in air was observed, which might affect the leaching behaviour of a monazite-type waste form in the long term [110,111].

## 5. Conclusions

Synthetic single phase SmPO_4_ with a monazite structure was irradiated with multienergy Au ions (1, 3.5 and 7 MeV) to investigate the response of potential SmPO_4_-based waste forms to internal *α*-decay of incorporated actinides. The threshold displacement energies *E*_d_ for monazite-structured SmPO_4_ to estimate dpa values were derived from atomistic simulations, revealing distinct differences to default *E*_d_ values used in SRIM/TRIM and those for zircon, which were used as surrogate values for monazite-type *Ln*PO_4_ in the past. The different displacement threshold energies used for the calculation of amorphisation doses in terms of dpa in the past impede the comparison to other ion irradiation experiments. However, the comparison of fluences required for amorphisation indicated a similar resistance of SmPO_4_ to ion-beam irradiation as observed for other *Ln*PO_4_ compounds with monazite structures. The irradiation effects on the local structure of SmPO_4_ revealed by the Raman spectra supports the assumption that amorphisation of the monazite structure is mainly due to a breaking of *Ln*–O bonds and increasing structural disorder in the local environment of the phosphate groups, with the phosphate tetrahedra behaving as tight units under irradiation. Moreover, fast recombination in case of displacement of oxygen atoms from the PO_4_ units is expected.

Although monazite-structured SmPO_4_ can be amorphised in ion-beam experiments at repository-relevant temperatures, annealing effects were observed already during the irradiation experiment itself, leading to partly crystalline domains even in the sample irradiated at the highest fluence. This suggests that a SmPO_4_ waste form for actinides will probably not undergo a macroscopic crystalline to amorphous phase transition. Moreover, with respect to amorphisation effects in nuclear waste forms in the repository environment due to *α*-decay of incorporated actinides, one should be aware that the dose rates (e.g., in dpa per s) are distinctively lower than in ion-beam irradiation experiments, i.e., the ratio between damage production and recovery/annealing is different, and further mechanisms such as *α*-radiation-induced annealing may contribute to the radiation resistance of monazite-type waste forms.

## Figures and Tables

**Table 1:** Buckingham potential parameters used in the simulations.

	*A*/eV	*B*/Å^–1^	*C*/Å^6^ eV
Sm–O	14,794.52	0.26	0.00
P–O	877.3	0.3594	0.0000
O–O	22,764.3	0.1390	27.879

**Table 2:** Structural data of monazite-type SmPO_4_ obtained by Rietveld refinement compared to selected published crystal data.

Reference	*a*/Å	*b*/Å	*c*/Å	*β*/°	*V*/Å^3^
This work	6.69035 (4)	6.89207 (4)	6.37219 (4)	103.8632 (4)	285.2647 (3)
[24]	6.6818 (12)	6.8877 (9)	6.3653 (9)	103.86 (1)	284.42
[87]	6.6891 (3)	6.8958 (3)	6.3770 (6)	103.9 (1)	285.54
[88]	6.6902 (9)	6.8935 (5)	6.3714 (3)	103.871 (9)	285.30
[18]	6.6888 (3)	6.8948 (2)	6.3725 (3)	103.87 (1)	285.32 (5)
[89]	6.803	6.961	6.394	104.2	293.52
[82] *	6.731	6.923	6.383	104.081	288.523
[67] *	6.686	6.880	6.344	104.032	283.128

* Values derived from atomistic simulations—due to the approximate character of the applied DFT method, the expected relative error in these values is up to about 3%.

**Table 3:** Average energy deposition and doses during triple-energy irradiation of SmPO_4_ with Au ions (1, 3.5, 7 MeV).

Fluence/Ions cm^−2^		Average Energy Deposition/keV cm^−3^			Average Dose/MGy		
		Total	Nuclear	Electronic	Total	Nuclear	Electronic
F1	6.0 × 10^12^	1.57 × 10^20^	4.85 × 10^19^	1.09 × 10^20^	4.8	1.5	3.3
F2	1.8 × 10^13^	4.72 × 10^20^	1.45 × 10^20^	3.26 × 10^20^	14.3	4.4	9.9
F3	5.1 × 10^14^	1.34 × 10^22^	4.12 × 10^21^	9.24 × 10^21^	403.9	124.5	279.4

**Table 4:** Results of SRIM/TRIM simulation of external Au ion irradiation of monazite-structured SmPO_4_ pellets and lamellae.

	Pellet Samples			Lamellae		
	F1	F2	F3	F1	F2	F3
Fluence/ions cm^−2^	6.0 × 10^12^	1.8 × 10^13^	5.1 × 10^14^	6.1 × 10^12^	1.8 × 10^13^	5.1 × 10^14^
dpa_(max)_	0.06	0.19	5.44	0.06	0.19	5.44
dpa_(avg)_	0.03 *	0.08 *	2.13 *	0.05	0.15	3.81

* Averaged over the irradiation-affected depth of 1640 nm.

**Table 5:** Position and FWHM of the Raman band *ν*_1_ of unirradiated and irradiated SmPO_4_ pellets and lamellae.

	Pellet Samples				Lamellae			
		F1	F2	F3		F1	F2	F3
Fluence/ions cm^−2^	0	6.0 × 10^12^	1.8 × 10^13^	5.1 × 10^14^	0	6.1 × 10^12^	1.8 × 10^13^	5.1 × 10^14^
*ν*_1_/cm^−1^	982.0	981.8	981.6	982.4	980.9	979.6	979.3	961.8
FWHM/cm^−1^	4.3	3.6	3.5	3.4	3.5	5.2	6.5	>>10

**Table 6:** Threshold displacement energies (*E*_d_ values) used in various studies on radiation effects in monazite-type phosphates (Σ: sum of *E*_d_ counting the value for O fourfold).

Phase	*E*_d_/eV				Used by	Comment
	*Ln*	P	O	Σ		
Monazite (natural)	25	25	25	150	[44]	
Monazite (natural)	79	23	47	290	[39]	analogy to ZrSiO_4_ [4]
*Ln*PO_4_ (*Ln*: La—Lu)	20	20	20	120	[46]	
LaPO_4_	20	20	20	120	[45]	
LaPO_4_	79	23	47	290	[39,47]	analogy to ZrSiO_4_ [4]
LaPO_4_	25	25	25	150	[50]	
LaPO_4_	56	75	8	163	[68]	derived from MD simulations
CePO_4_	25	25	28	162	[42,49]	SRIM/TRIM default [77]
SmPO_4_	49	65	8	146	this work	derived from MD simulations
GdPO_4_	51	75	8	158	[68]	derived from MD simulations

**Table 7:** Comparison of alpha doses (*D*_alpha_) and alpha dose rates incurred in Pu waste forms (10 wt% Pu loading) in 10^6^ years to nonmetamict monazites from nature and alpha-doped *Ln*-phosphates with monazite structure (in parentheses: exposure time (*Ln*PO_4_) or age (monazites), respectively).

Material	*D*_alpha_/*α* g^−1^	Dose Rate/*α* g^−1^ s^−1^	Source
Pu wasteform (10 wt% Pu)	1 × 10^20^ to 4 × 10^20^ (1 Ma)	~1 × 10^7^	this work (cf. Table S4)
Monazite-(Sm) (natural)	2.9 × 10^20^ to 3.7 × 10^20^ (2640 Ma)	3471 to 4492	[43]
Monazite-(Ce) (natural)	2.43 × 10^19^ (474 to 479 Ma)	1618	[33]
Monazites (natural)	1.3 × 10^18^ to 1.7 × 10^20^ (24 to 1928 Ma)	1718 to 3561	[34]
Monazites (natural)	6.0 × 10^18^ to 1.1 × 10^20^ (101 to 1391 Ma)	379 to 4973	[36]
Monazite-(Ce) (natural)	2.4 × 10^19^ to 1.7 × 10^20^ (500 to 1928 Ma)	1522 to 2796	[35]
^244^Cm-doped LuPO_4_	5.0 × 10^19^ (18 a)	8.8 × 10^10^	[103]
^238^Pu-doped (La,Pu)PO_4_	2.5 × 10^18^		[101]
^238^Pu-doped (La,Ca,Th)PO_4_	7.5 × 10^18^ (5 a)	4.7 × 10^10^	[50]

## Data Availability

The data presented in this study are available on request from the corresponding author.

## Supplementary Materials

The following supporting information can be downloaded at: https://www.mdpi.com/article/10.3390/ma15103434/s1, Figure S1: SEM-BSE images of polished SmPO_4_ pellets—the uniform grey values indicate the chemical homogeneity of the synthesised monazite-structured SmPO_4_; Table S1: alpha decay events in the decay chains of ^238^Pu to ^242^Pu and fractional contributions of specific decay events to the total alpha dose for selected periods (half-lives: y: years, d: days, h: hours, m: minutes, s: seconds; total alpha doses per gram of initial Pu isotope; nuclear data from ENDF/B-VIII.0, Brown et al. [102]). Energies refer to decay events with emission probabilities exceeding 5%; Table S2: Results of Monte Carlo (MC) simulations (SRIM/TRIM) of alpha-event effects in monazite-structured SmPO_4_. In the respective decay chains, only decay events contributing to more than 1% of the total alpha dose received in one million years are considered. The displacements per decay event are weighted averages for the respective alpha and recoil energies given in Table S1; TableS3: Isotopic composition of Pu produced in nuclear reactors for different burn-up levels and reactor designs (HWR: Heavy water reactor; AGR: Advanced gas-cooled reactor, BWR: Boiling-water reactor, PWR: Pressurised-water reactor, VVER: Pressurised water-water energetic reactor, MOX: mixed oxide (U,Pu)O_2_ fuel; n.a.: information not available—isotope produced but only in small amounts); Table S4: Calculated (integral) alpha doses (*D*_alpha_) and dose rates for waste forms containing 10 wt% Pu waste load originating from different sources (Pu isotopic composition from Table S3) compared to ^239^Pu. References [112,113,114,115] are cited in the Supplementary Materials.

## References

[1] Ringwood A.E., Kesson S.E., Reeve K.D., Levins D.M., Ramm E.J., Lutze W., Ewing R.C. (1988). Synroc. Radioactive Waste Forms for the Future.

[2] Harker A.B., Lutze W., Ewing R.C. (1988). Tailored Ceramics. Radioactive Waste Forms for the Future.

[3] Ewing R., Weber W., Clinard F. (1995). Radiation effects in nuclear waste forms for high level radioactive waste. Prog. Nucl. Energy.

[4] Weber W.J., Ewing R.C., Catlow C.R.A., de la Rubia T.D., Hobbs L.W., Kinoshita C., Matzke H., Motta A.T., Nastasi M., Salje E.K.H. (1998). Radiation effects in crystalline ceramics for the immobilization of high-level nuclear waste and plutonium. J. Mater. Res..

[5] Ewing R.C. (1999). Nuclear waste forms for actinides. Proc. Natl. Acad. Sci. USA.

[6] Lumpkin G.R. (2006). Ceramic waste forms for actinides. Elements.

[7] Ewing R.C. (2007). Ceramic matrices for plutonium disposition. Prog. Nucl. Energy.

[8] Weber W.J., Navrotsky A., Stefanovsky S., Vance E.R., Vernaz E. (2009). Materials science of high-level nuclear waste immobilization. MRS Bull..

[9] Ewing R.C. (2011). Actinides and radiation effects: Impact on the back-end of the nuclear fuel cycle. Mineral. Mag..

[10] Deissmann G., Neumeier S., Modolo G., Bosbach D. (2012). Durability of potential plutonium wasteforms under repository conditions. Mineral. Mag..

[11] Boatner L., Beall G., Abraham M., Finch C., Huray P., Rappaz M. (1980). Scientific Basis for Nuclear Waste Management.

[12] Boatner L., Sales B., Lutze W., Ewing R.C. (1988). Monazite. Radioactive Waste Forms for the Future.

[13] Ewing R.C., Wang L. (2002). Phosphates as nuclear waste forms. Rev. Mineral. Geochem..

[14] Terra O., Dacheux N., Audubert F., Podor R. (2006). Immobilization of tetravalent actinides in phosphate ceramics. J. Nucl. Mater..

[15] Oelkers E., Montel J.M. (2008). Phosphates and nuclear waste storage. Elements.

[16] Clavier N., Podor R., Dacheux N. (2011). Crystal chemistry of the monazite structure. J. Eur. Ceram. Soc..

[17] Dacheux N., Clavier N., Podor R. (2013). Monazite as a promising long-term radioactive waste matrix: Benefits of high-structural flexibility and chemical durability. Am. Mineral..

[18] Schlenz H., Heuser J., Neumann A., Schmitz S., Bosbach D. (2013). Monazite as a suitable actinide waste form. Cryst. Mater..

[19] Heuser J., Bukaemskiy A., Neumeier S., Neumann A., Bosbach D. (2014). Raman and infrared spectroscopy of monazite-type ceramics used for nuclear waste conditioning. Prog. Nucl. Energy.

[20] Neumeier S., Arinicheva Y., Ji Y., Heuser J.M., Kowalski P.M., Kegler P., Schlenz H., Bosbach D., Deissmann G. (2017). New insights into phosphate based materials for the immobilisation of actinides. Radiochim. Acta.

[21] Schlenz H., Neumeier S., Hirsch A., Peters L., Roth G. (2017). Highlights in Applied Mineralogy.

[22] Arinicheva Y. (2019). Monazite-Type Ceramics as Nuclear Waste Form: Crystal Structure, Microstructure and Properties.

[23] Ji Y., Kowalski P.M., Kegler P., Huittinen N., Marks N.A., Vinograd V.L., Arinicheva Y., Neumeier S., Bosbach D. (2019). Rare-earth orthophosphates from atomistic simulations. Front. Chem..

[24] Ni Y., Hughes J.M., Mariano A.N. (1995). Crystal-chemistry of the monazite and xenotime structures. Am. Mineral..

[25] Boatner L.A. (2002). Synthesis, structure and properties of monazite, pretulite, and xenotime. Rev. Mineral. Geochem..

[26] Montel J.-M., Devidal J.-L., Avignant D. (2002). X-ray diffraction study of brabantite-monazite solid solution. Chem. Geol..

[27] Ewing R.C., Meldrum A., Wang L., Wang S. (2000). Radiation-induced amorphization. Rev. Mineral. Geochem..

[28] Ewing R.C., Weber W.J. (2010). The Chemistry of the Actinide and Transactinide Elements.

[29] Meldrum A., Cherniak D. (2009). Materials science with ion beams. Topics in Applied Physics.

[30] Grammacioli C.M., Segalstad T.V. (1978). A uranium- and thorium-rich monazite from a south-alpine pegmatite at Piona, Italy. Am. Mineral..

[31] Lumpkin G.R. (1998). Rare-element mineralogy and internal evolution of the Rutherford #2 pegmatite, Amelia County, Virginia: A classic locality revisited. Can. Mineral..

[32] Hoshino M., Sanematsu K., Watanabe Y. (2016). REE mineralogy and resources. Handb. Phys. Chem. Rare Earths.

[33] Seydoux-Guillaume A.-M., Wirth R., Nasdala L., Gottschalk M., Montel J.M., Heinrich W. (2002). An XRD, TEM and Raman study of experimentally annealed natural monazite. Phys. Chem. Miner..

[34] Seydoux-Guillaume A.-M., Wirth R., Deutsch A., Schärer U. (2004). Microstructure of 24-1928 Ma concordant monazites: Implications for geochronology and nuclear waste deposits. Geochim. Cosmochim. Acta.

[35] Seydoux-Guillaume A.-M., David M.-L., Alix K., Datas L., Bingen B. (2016). Trapping of helium in nano-bubbles in euxenite: Positive identification and implications. Earth Planet. Sci. Lett..

[36] Ruschel K., Nasdala L., Kronz A., Hanchar J., Többens D., Skoda R., Finger F., Möller A. (2012). A Raman spectroscopic study on the structural disorder of monazite-(Ce). Miner. Petrol..

[37] Ewing R.C., Haaker R.F. (1980). The metamict state: Implications for radiation damage in crystalline waste forms. Nucl. Chem. Waste Manag..

[38] Black L.P., Fitzgerald J.D., Harley S.L. (1984). Pb isotopic composition, colour, and microstructure of monazites from a polymetamorphic rock in Antarctica. Contrib. Mineral. Petrol..

[39] Meldrum A., Boatner L.A., Weber W.J., Ewing R.C. (1998). Radiation damage in zircon and monazite. Geochim. Cosmochim. Acta.

[40] Montel J.-M., Razafimahatratra D., de Parseval P., Poitrasson F., Moine B., Seydoux-Guillaume A.-M., Pik R., Arnaud N., Gibert F. (2018). The giant monazite crystals from Manangotry (Madagascar). Chem. Geol..

[41] Seydoux-Guillaume A.-M., Deschanels X., Baumier C., Neumeier S., Weber W.J., Peuget S. (2018). Why natural monazite never becomes amorphous: Experimental evidence for alpha self-healing. Am. Mineral..

[42] Nasdala L., Akhmadaliev S., Artac A., Chanmuang N.C., Habler G., Lenz C. (2018). Irradiation effects in monazite–(Ce) and zircon: Raman and photoluminescence study of Au-irradiated FIB foils. Phys. Chem. Miner..

[43] Masau M., Černý P., Cooper M.A., Chapman R. (2002). Monazite-(Sm), a new member of the monazite group from the Annie Claim #3 granitic pegmatite, southwestern Manitoba. Can. Mineral..

[44] Meldrum A., Wang L.M., Ewing R.C. (1996). Ion beam induced amorphization of monazite. Nucl. Instrum. Meth. B.

[45] Meldrum A., Boatner L.A., Wang L.M., Ewing R.C. (1997). Ion-beam-induced amorphization of LaPO_4_ and ScPO_4_. Nucl. Instrum. Meth. B.

[46] Meldrum A., Boatner L.A., Ewing R.C. (1997). Displacive radiation effects in the monazite- and zircon-structure orthophosphates. Phys. Rev. B.

[47] Meldrum A., Boatner L.A., Ewing R.C. (2000). A comparison of radiation effects in crystalline ABO_4_-type phosphates and silicates. Mineral. Mag..

[48] Picot V., Deschanels X., Peuget S., Glorieux B., Seydoux-Guillaume A.-M., Wirth R. (2008). Ion beam radiation effects in monazite. J. Nucl. Mater..

[49] Nasdala L., Grotzschel R., Probst S., Bleisteiner B. (2010). Irradiation damage in Monazite-(Ce): An example to establish the limits of Raman confocality and depth resolution. Can. Mineral..

[50] Deschanels X., Seydoux-Guillaume A.-M., Magnin V., Mesbah A., Tribet M., Moloney M., Serruys Y., Peuget S. (2014). Swelling induced by alpha decay in monazite and zirconolite ceramics: A XRD and TEM comparative study. J. Nucl. Mater..

[51] Karioris F.G., Gowda K.A., Cartz L. (1981). Heavy ion bombardement of monoclinic ThSiO_4_, ThO_2_ and monazite. Radiat. Eff. Lett..

[52] Ehlert T.C., Gowda K.A., Karioris F.G., Cartz L. (1983). Differential scanning calorimetry of heavy ion bombarded synthetic monazite. Radiat. Eff..

[53] Meldrum A., Boatner L.A., Ewing R.C. (1997). Electron-irradiation-induced nucleation and growth in amorphous LaPO_4_, ScPO_4_, and zircon. J. Mater. Res..

[54] Seydoux-Guillaume A.-M., Wirth R., Ingrin J. (2007). Contrasting response of ThSiO_4_ and monazite to natural irradiation. Eur. J. Mineral..

[55] Shannon R.D. (1976). Revised effective ionic radii and systematic studies of interatomic distances in halides and chalcogenides. Acta Crystallogr..

[56] Heuser J.M. (2015). Keramiken des Monazit-Typs zur Immobilisierung von Minoren Actinoiden und Plutonium.

[57] Heuser J.M., Palomares R.I., Bauer J.D., Lozano Rodriguez J.M., Cooper J., Lang M., Scheinost A.C., Schlenz H., Winkler B., Bosbach D. (2018). Structural characterisation of (Sm,Tb)PO_4_ solid solutions and pressure-induced phase transitions. J. Eur. Ceram. Soc..

[58] Kinchin G.H., Pease R.S. (1955). The displacement of atoms in solids by radiation. Rep. Prog. Phys..

[59] Brinkman J.A. (1956). Production of atomic displacements by high-energy particles. Am. J. Phys..

[60] Nastasi M., Mayer J.W., Hirvonen J.K. (1996). Ion-Solid Interactions—Fundamentals and Applications.

[61] Smith R. (1997). Atomic and Ion Collisions in Solids and at Surfaces: Theory, Simulation and Applications.

[62] Biersack J.P., Haggmark L.G. (1980). A Monte Carlo computer program for the transport of energetic ions in amorphous targets. Nucl. Instrum. Meth..

[63] Ziegler J.F., Biersack J.P., Ziegler M.D. (2008). SRIM: The Stopping and Range of Ions in Matter.

[64] Lunéville L., Simeone D., Jouanne C. (2006). A new tool to compare neutron and ion irradiation in materials. Nucl. Instrum. Meth. B.

[65] Lunéville L., Simeone D., Jouanne C. (2006). Calculation of radiation damage induced by neutrons in compound materials. J. Nucl. Mater..

[66] Lunéville L., Simeone D. (2016). DART, a BCA code to assess and compare primary irradiation damage in nuclear materials submitted to neutron and ion flux. EPJ Web Conf..

[67] Li Y., Kowalski P.M., Beridze G., Blanca-Romero A., Ji Y., Vinograd V.L., Gale J., Bosbach D. (2016). Atomistic simulations of ceramic materials relevant for nuclear waste management: Cases of monazite and pyrochlore. Ceram. Trans..

[68] Ji Y., Kowalski P.M., Neumeier S., Deissmann G., Kulriya P.K., Gale J.D. (2017). Atomistic modeling and experimental studies of radiation damage in monazite-type LaPO_4_ ceramics. Nucl. Instrum. Meth. B.

[69] Boakye E.E., Mogilevsky P., Hay R.S., Fair G.E. (2008). Synthesis and phase composition of lanthanide phosphate nanoparticles *Ln*PO_4_ (*Ln* = La, Gd, Tb, Dy, Y) and solid solutions for fiber coatings. J. Am. Ceram. Soc..

[70] Rietveld H.M. (1967). Line profiles of neutron powder-diffraction peaks for structure refinement. Acta Crystallogr..

[71] Coelho A. (2007). TOPAS Academic User Manual.

[72] Cheary R.W., Coelho A. (1992). A fundamental parameters approach to x-ray line-profile fitting. J. Appl. Crystallogr..

[73] Dijkman F.G., van der Maas J.H. (1976). Dependence of bandshape and depolarization ratio on slitwidth. Appl Spectrosc..

[74] Irmer G. (1985). Zum Einfluss der Apparatefunktion auf die Bestimmung von Streuquerschnitten und Lebensdauern aus optischen Phononenspektren. Exp. Tech. Phys..

[75] Nasdala L., Wenzel M., Vavra G., Irmer G., Wenzel T., Kober B. (2001). Metamictisation of natural zircon: Accumulation versus thermal annealing of radioactivity-induced damage. Contrib. Mineral. Petr..

[76] Váczi T. (2014). A new, simple approximation for the deconvolution of instrumental broadening in spectroscopic band profiles. Appl. Spectrosc..

[77] Ziegler J.F., Ziegler M.D., Biersack J.P. (2010). SRIM—The stopping and range of ions in matter (2010). Nucl. Instrum. Meth. B.

[78] Robinson M.T. (1994). The binary collision approximation: Background and introduction. Radiat. Eff. Defects Solids.

[79] Plimpton S. (1995). Fast parallel algorithms for short-range molecular dynamics. J. Comput. Phys..

[80] Robinson M., Marks N.A., Whittle K.R., Lumpkin G.R. (2012). Systematic calculation of threshold displacement energies: Case study in rutile. Phys. Rev. B.

[81] Buckingham R.A. (1938). The classical equation of state of gaseous helium, neon and argon. Proc. R. Soc. Lond. A.

[82] Blanca-Romero A., Kowalski P.M., Beridze G., Schlenz H., Bosbach D. (2014). Performance of DFT+U method for prediction of structural and thermodynamic parameters of monazite-type ceramics. J. Comput. Chem..

[83] Gale J.D., Henson N.J. (1994). Derivation of interatomic potentials for microporous aluminophosphates from the structure and properties of berlinite. J. Chem. Soc. Faraday Trans..

[84] Girard S., Gale J.D., Mellot-Draznieks C., Ferey G. (2001). Derivation of interatomic potentials for gallophosphates from the GaPO_4_−quartz structure: Transferability study to gallosilicates and zeotype gallophosphates. Chem. Mater..

[85] Robinson M., Marks N.A., Lumpkin G.R. (2012). Sensitivity of the threshold displacement energy to temperature and time. Phys. Rev. B.

[86] Mullica D.F., Grossie D.A., Boatner L.A. (1985). Coordination geometry and structural determinations of samarium orthophosphate, europium orthophosphate, and gadolinium orthophosphate. Inorg. Chim. Acta.

[87] Ushakov S., Helean K., Navrotsky A., Boatner L. (2001). Thermochemistry of rare-earth orthophosphates. J. Mater. Res..

[88] Popa K., Konings R. (2006). High-temperature heat capacities of EuPO_4_ and SmPO_4_ synthetic monazites. Thermochim. Acta.

[89] Rustad J.R. (2012). Density functional calculations of the enthalpies of formation of rare-earth orthophosphates. Am. Mineral..

[90] Perdew J.P., Ruzsinszky A., Csonka G.I., Vydrov O.A., Scuseria G.E., Constantin L.A., Zhou X., Burke K. (2008). Restoring the density-gradient expansion for exchange in solids and surfaces. Phys. Rev. Lett..

[91] Kowalski P.M., Ji Y., Li Y., Arinicheva Y., Beridze G., Neumeier S., Bukaemskiy A., Bosbach D. (2017). Simulation of ceramic materials relevant for nuclear waste management: Case of La_1-x_Eu_x_PO_4_ solid solution. Nucl. Instrum. Meth. B.

[92] Gouadec G., Colomban P. (2007). Raman spectroscopy of nanomaterials: How spectra relate to disorder, particle size and mechanical properties. Prog. Cryst. Growth Charact. Mater..

[93] Begun G.M., Beall G.W., Boatner L.A., Gregor W.J. (1981). Raman spectra of the rare earth orthophosphates. J. Raman Spectrosc..

[94] Silva E., Ayala A., Guedes I., Paschoal C., Moreira R., Loong C.-K., Boatner L. (2006). Vibrational spectra of monazite-type rare-earth orthophosphates. Opt. Mater..

[95] Salje E. (1973). Experimentelle Untersuchung der Ramanstreuung an Kristallpulvern. J. Appl. Cryst..

[96] Shimizu R., Ogasawara Y. (2014). Radiation damage to Kokchetav UHPM diamonds in zircon: Variations in Raman, photoluminescence, and cathodoluminescence spectra. Lithos.

[97] Popovic L., de Waal D., Boeyens J.C.A. (2005). Correlation between Raman wavenumbers and P-O bond lengths in crystalline inorganic phosphates. J. Raman Spectrosc..

[98] Williford R.E., Devanathan R., Weber W.J. (1998). Computer simulation of displacement energies for several ceramic materials. Nucl. Instrum. Meth. B.

[99] Park B., Weber W.J., Corrales L.R. (2001). Molecular-dynamics simulation study of threshold displacements and defect formation in zircon. Phys. Rev. B.

[100] Lang M., Zhang F., Ewing R., Lian J., Trautmann C., Wang Z. (2009). Structural modifications of Gd_2_Zr_2-x_Ti_x_O_7_ pyrochlore induced by swift heavy ions: Disordering and amorphization. J. Mater. Res..

[101] Burakov B.E., Yagovkina M.A., Garbuzov V.M., Kitsay A.A., Zirlin V.A. (2004). Self-irradiation of monazite ceramics: Contrasting behavior of PuPO_4_ and (La,Pu)PO_4_ doped with Pu-238. Mater. Res. Soc. Symp. Proc..

[102] Brown D.A., Chadwick M.B., Capote R., Kahler A.C., Trkov A., Herman M.W., Sonzogni A.A., Danon Y., Carlson A.D., Dunn M. (2018). ENDF/B-VIII.0: The 8th major releae of the Nuclear Reaction Data Library with CIELO-project cross sections, new standards and thermal scattering data. Nucl. Data Sheets.

[103] Luo J.S., Liu G.K. (2001). Microscopic effects of self-radiation damage in ^244^Cm-doped LuPO_4_ crystals. J. Mater. Res..

[104] Nasdala L., Grambole D., Ruschel K. (2013). Review of effects of radiation damage on the luminescence emission of minerals, and the example of He-irradiated CePO_4_. Mineral. Petrol..

[105] Hyatt N.C. (2017). Plutonium management policy in the United Kingdom: The need for a dual track strategy. Energy Policy.

[106] Arinicheva Y., Popa K., Scheinost A.C., Rossberg A., Dieste-Blanco O., Raison P., Cambriani A., Somers J., Bosbach D., Neumeier S. (2017). Structural investigations of (La,Pu)PO_4_ monazite solid solutions: XRD and XAFS study. J. Nucl. Mater..

[107] International Atomic Energy Agency (IAEA) (1998). Safe Handling and Storage of Plutonium.

[108] Nasdala L., Akhmadaliev S., Burakov B.E., Chanmuang N.C., Škoda R. (2020). The absence of metamictisation in natural monazite. Sci. Rep..

[109] Mir A.H., Peuget S. (2020). Using external ion irradiations for simulating self-irradiation damage in nuclear waste glasses: State of the art, recommendations and prospects. J. Nucl. Mater..

[110] Shiryaev A.A., Burakov B.E., Nickolsky M.S., Yapaskurt V.O., Pavlushin A.D., Grigoriev M.S., Vlasova I.E. (2020). Surface features on aged ^238^Pu-doped Eu-monazite. Radiochim. Acta.

[111] Shiryaev A.A., Burakov B.E., Yapaskurt V.O., Egorov A.V., Vlasova I.E. (2020). Microstructure of aged ^238^Pu-doped La-monazite ceramic and peculiarities of its X-ray emission spectra. MRS Adv..

[112] OECD-NEA (1989). Plutonium Fuel: An Assessment.

[113] Wallenius M., Peerani P., Koch L. (2000). Origin determination of plutonium material in nuclear forensics. J. Radioanal. Nucl. Chem..

[114] OECD-NEA (1995). Physics of Plutonium Recycling, Vol. 1—Issues and Perspectives.

[115] Kang J., von Hippel F.N., MacFarlane A., Nelson R. (2002). Storage MOX: A third way for plutonium disposal?. Sci. Glob. Secur..

