# Supplementary material for: Monazite-Type SmPO4 as Potential Nuclear Waste Form: Insights into Radiation Effects from Ion-Beam Irradiation and Atomistic Simulations
Source: Materials (Basel). 2022 May 10;15(10):3434. doi: 10.3390/ma15103434 (PMC9146725; doi:10.3390/ma15103434)
Supplement: Supplementary file 1 [file materials-15-03434-s001.zip › materials-1703868-supplementary.pdf]

Supplementary Material

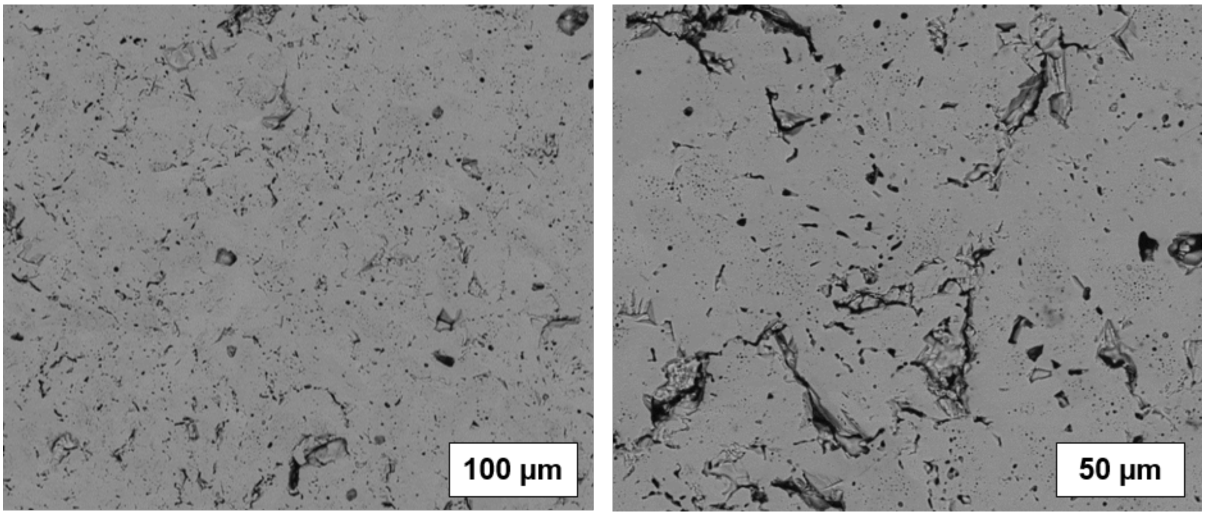

Figure S1. SEM-BSE images of polished $SmPO_4$ pellets—the uniform grey values indicate the chemical homogeneity of the synthesised monazite-structured $SmPO_4$.

Table S1. Alpha decay events in the decay chains of ^{238}Pu to ^{242}Pu and fractional contributions of specific decay events to the total alpha dose for selected periods (half-lives: y: years, d: days, h: hours, m: minutes, s: seconds; total alpha doses per gram of initial Pu isotope; nuclear data from ENDF/B-VIII.0, Brown et al. [102]). Energies refer to decay events with emission probabilities exceeding 5%.

**^{238}Pu decay chain**								
**Event**	**Half-life**	**Emission probability / %**	**Energy / keV**		**Fraction of alpha decays in ... years / %**			
			**alpha**	**recoil**	**10^3 y**	**10^4 y**	**10^5 y**	**10^6 y**
$^{238}Pu \rightarrow {}^{234}U$	87.7 y	70.9	5499	94.0	99.8	96.9	56.9	13.5
		29.0	5456	93.3				
$^{234}U \rightarrow {}^{230}Th$	245,500 y	71.4	4775	83.1	0.3	2.7	14.0	12.7
		28.4	4722	82.2				
$^{230}Th \rightarrow {}^{226}Ra$	75,380 y	76.3	4687	83.0	<0.1	0.1	5.0	12.3
		23.4	4621	81.8				
$^{226}Ra \rightarrow {}^{222}Rn$	1,600 y	94.5	4784	86.3	<0.1	<0.1	4.8	12.3
		5.6	4601	82.9				
$^{222}Rn \rightarrow {}^{218}Po$	3.8235 d	99.9	5490	100.8	<0.1	<0.1	4.8	12.3
$^{218}Po \rightarrow {}^{214}Pb$	3.098 m	100.0	6002	112.3	<0.1	<0.1	4.8	12.3
$^{214}Po \rightarrow {}^{210}Pb$	164.3 µs	100.0	7687	146.5	<0.1	<0.1	4.8	12.3
$^{210}Po \rightarrow {}^{206}Pb$	138.376 d	100.0	5304	103.1	<0.1	<0.1	4.8	12.3
alpha dose / α g^{-1}					$2.5\cdot10^{21}$	$2.6\cdot10^{21}$	$4.4\cdot10^{21}$	$1.9\cdot10^{22}$

^{239}Pu decay chain

**Event**	**Half-life**	**Emission probability / %**	**Energy / keV**		**Fraction of alpha decays in ... years / %**			
			**alpha**	**recoil**	**10^3 y**	**10^4 y**	**10^5 y**	**10^6 y**
$^{239}Pu \rightarrow {}^{235}U$	24,110 y	70.8	5157	87.8	100.0	100.0	99.9	99.4
		17.1	5144	87.6				
		11.9	5106	86.9				
$^{235}U \rightarrow {}^{231}Th$	703.8 My	5.0	4596	79.6	<0.1	<0.1	<0.1	<0.1
		55.0	4398	76.2				
		17.0	4366	75.6				
		5.7	4215	73.0				
$^{231}Pa \rightarrow {}^{227}Ac$	32,760 y	11.0	5059	89.2	<0.1	<0.1	<0.1	<0.1
		20.0	5028	88.7				
		25.4	5014	88.4				
		22.8	4951	87.3				
		8.4	4736	83.5				
$^{227}Th \rightarrow {}^{223}Ra$	18.68 d	24.2	6038	108.4	<0.1	<0.1	<0.1	<0.1
		23.5	5978	107.3				
		20.4	5757	103.3				
		8.3	5709	102.5				
$^{223}Ra \rightarrow {}^{219}Rn$	11.43 d	9.0	5747	105.0	<0.1	<0.1	<0.1	<0.1
		51.6	5716	104.5				
		25.2	5607	102.5				
		9.0	5540	101.2				
$^{219}Rn \rightarrow {}^{215}Po$	3.96 s	79.4	6819	126.9	<0.1	<0.1	<0.1	<0.1
		12.9	6553	122.0				
		7.5	6425	119.6				
$^{215}Po \rightarrow {}^{211}Pb$	1.781 ms	100.0	7386	140.1	<0.1	<0.1	<0.1	<0.1
$^{211}Bi \rightarrow {}^{207}Tl$	2.14 m	83.8	6623	128.1	<0.1	<0.1	<0.1	<0.1
		16.2	6278	121.4				
alpha dose / α g^{-1}					$7.1 \cdot 10^{19}$	$6.3 \cdot 10^{20}$	$2.4 \cdot 10^{21}$	$2.5 \cdot 10^{21}$

^{240}Pu decay chain

Event	Half-life	Emission probability / %	Energy / keV		Fraction of alpha decays in ... years / %			
			alpha	recoil	103 y	104 y	105 y	106 y
$^{240}Pu \rightarrow {}^{236}U$	6,561 y	72.8	5168	87.6	100.0	100.0	99.7	97.2
		27.1	5124	86.9				
$^{236}U \rightarrow {}^{232}Th$	23.42 My	73.8	4494	77.5	<0.1	<0.1	0.3%	2.8
		25.9	4445	76.7				
$^{232}Th \rightarrow {}^{228}Ra$	14.05 Gy	78.2	4012	70.4	<0.1	<0.1	<0.1	<0.1
		21.7	3947	69.3				
$^{228}Th \rightarrow {}^{224}Ra$	1.912 y	72.2	5423	96.9	<0.1	<0.1	<0.1	<0.1
		27.2	5340	95.4				
$^{224}Ra \rightarrow {}^{220}Rn$	3.66 d	94.9	5685	103.4	<0.1	<0.1	<0.1	<0.1
		5.1	5449	99.1				

$^{220}Rn \rightarrow {}^{216}Po$	55.6 s	99.9	6288	116.5	<0.1	<0.1	<0.1	<0.1
$^{216}Po \rightarrow {}^{212}Pb$	145 ms	100.0	6778	128.0	<0.1	<0.1	<0.1	<0.1
$^{212}Bi \rightarrow {}^{208}Tl^{a}$	1.009 h	27.1	6090	117.2	<0.1	<0.1	<0.1	<0.1
		69.9	6051	116.4				
$^{212}Po \rightarrow {}^{208}Pb$	299 ns	100.0	8784	169.1	<0.1	<0.1	<0.1	<0.1
alpha dose / α g^{-1}					$2.5 \cdot 10^{20}$	$1.6 \cdot 10^{21}$	$2.5 \cdot 10^{21}$	$2.6 \cdot 10^{21}$

[a] Branching decay: $^{212}Bi \rightarrow {}^{208}Tl$ (35.9%), $^{212}Bi \rightarrow {}^{212}Po \rightarrow {}^{208}Pb$ (64.1%)

^{241}Pu decay chain

Event	Half-life	Emission probability / %	Energy / keV		Fraction of alpha decays in ... years / %			
			alpha	recoil	10^3 y	10^4 y	10^5 y	10^6 y
$^{241}Am \rightarrow {}^{237}Np$	432.6 y	84.8	5486	92.6	100.0	99.7	94.2	38.8
		13.1	5443	91.9				
$^{237}Np \rightarrow {}^{233}Pa$	2.144 My	47.6	4788	82.2	<0.1	0.3	3.0	10.7
		23.2	4771	82.0				
		9.3	4767	81.9				
		6.4	4640	79.7				
$^{233}U \rightarrow {}^{229}Th$	159,200 y	84.3	4824	84.3	<0.1	<0.1	0.6	8.5
		13.2	4784	83.6				
$^{229}Th \rightarrow {}^{225}Ra$	7,340 y	6.6	5053	89.9	<0.1	<0.1	0.5	8.4
		6.0	4968	88.4				
		10.2	4901	87.2				
		56.2	4845	86.2				
		5.0	4838	86.1				
		9.3	4815	85.6				
$^{225}Ac \rightarrow {}^{221}Fr$	10.0 d	50.7	5830	105.6	<0.1	<0.1	0.5	8.4
		18.1	5793	104.9				
		8.6	5791	104.9				
		8.0	5732	103.8				
$^{221}Fr \rightarrow {}^{217}At$	4.9 m	83.4	6341	117.0	<0.1	<0.1	0.5	8.4
		15.1	6126	113.0				
$^{217}At \rightarrow {}^{213}Bi$	32.3 ms	99.9	7067	132.8	<0.1	<0.1	0.5	8.4
$^{213}Bi \rightarrow {}^{209}Tl^{a}$	45.59 m	93.0	5869	112.4	<0.1	<0.1	<0.1	0.2
		7.4	5549	106.3				
$^{213}Po \rightarrow {}^{209}Pb$	4.2 μs	100.0	8376	160.4	<0.1	<0.1	0.5	8.2
$^{209}Bi \rightarrow {}^{205}Tl$	1.9E+19 y	99.9	3077	60.1	<0.1	<0.1	<0.1	<0.1
alpha dose / α g^{-1}					$2.0 \cdot 10^{21}$	$2.5 \cdot 10^{21}$	$2.7 \cdot 10^{21}$	$6.4 \cdot 10^{21}$

[a] Branching decay: $^{213}Bi \rightarrow {}^{209}Tl$ (2.2%), $^{213}Bi \rightarrow {}^{213}Po \rightarrow {}^{209}Pb$ (97.8%)

^{242}Pu decay chain

Event	Half-life	Emission probability / %	Energy / keV		Fraction of alpha decays in ... years / %			
			alpha	recoil	10^3 y	10^4 y	10^5 y	10^6 y
$^{242}Pu \rightarrow {}^{238}U$	373,500 y	76.5	4902	82.4	100.0	100.0	100.0	100.0
		23.5	4858	81.7				

$^{238}U \rightarrow ^{234}Th$	4.468 Gy	79.0	4198	71.8	<0.1	<0.1	<0.1	<0.1
		20.9	4151	71.0				
$^{234}U \rightarrow ^{230}Th$	245,500 y	71.4	4775	83.1	<0.1	<0.1	<0.1	<0.1
		28.4	4722	82.2				
$^{230}Th \rightarrow ^{226}Ra$	75,380 y	76.3	4687	83.0	<0.1	<0.1	<0.1	<0.1
		23.4	4621	81.8				
$^{226}Ra \rightarrow ^{222}Rn$	1,600 y	94.5	4784	86.3	<0.1	<0.1	<0.1	<0.1
		5.6	4601	82.9				
$^{222}Rn \rightarrow ^{218}Po$	3.8235 d	99.9	5490	100.8	<0.1	<0.1	<0.1	<0.1
$^{218}Po \rightarrow ^{214}Pb$	3.098 m	100.0	6002	112.3	<0.1	<0.1	<0.1	<0.1
$^{214}Po \rightarrow ^{210}Pb$	164.3 μs	100.0	7687	146.5	<0.1	<0.1	<0.1	<0.1
$^{210}Po \rightarrow ^{206}Pb$	138.376 d	100.0	5304	103.1	<0.1	<0.1	<0.1	<0.1
alpha dose / α g^{-1}					$4.6 \cdot 10^{18}$	$4.6 \cdot 10^{19}$	$4.2 \cdot 10^{20}$	$2.1 \cdot 10^{21}$

Table S2. Results of Monte Carlo (MC) simulations (SRIM/TRIM) of alpha-event effects in monazite-structured $SmPO_4$. In the respective decay chains, only decay events contributing to more than 1% of the total alpha dose received in 1 million years are considered. The displacements per decay event are weighted averages for the respective alpha and recoil energies given in Table S1.

Decay event	Displacements (alpha)	Displacements (recoil)	Displacements (total)	dpa
**^{238}Pu decay chain**				
$^{238}Pu \rightarrow {}^{234}U$	445	2927	3372	$2.29\cdot10^{-19}$
$^{234}U \rightarrow {}^{230}Th$	450	2641	3092	$2.10\cdot10^{-19}$
$^{230}Th \rightarrow {}^{226}Ra$	449	2612	3062	$2.08\cdot10^{-19}$
$^{226}Ra \rightarrow {}^{222}Rn$	444	2709	3153	$2.14\cdot10^{-19}$
$^{222}Rn \rightarrow {}^{218}Po$	449	3118	3567	$2.42\cdot10^{-19}$
$^{218}Po \rightarrow {}^{214}Pb$	450	3462	3567	$2.66\cdot10^{-19}$
$^{214}Po \rightarrow {}^{210}Pb$	480	4385	4864	$3.30\cdot10^{-19}$
$^{210}Po \rightarrow {}^{206}Pb$	437	3192	3629	$2.46\cdot10^{-19}$
**^{239}Pu decay chain**				
$^{239}Pu \rightarrow {}^{235}U$	450	2758	3207	$2.18\cdot10^{-19}$
**^{240}Pu decay chain**				
$^{240}Pu \rightarrow {}^{236}U$	451	2748	3199	$2.17\cdot10^{-19}$
$^{236}U \rightarrow {}^{232}Th$	435	2482	2917	$1.98\cdot10^{-19}$
**^{241}Pu decay chain**				
$^{241}Am \rightarrow {}^{237}Np$	448	2898	3346	$2.27\cdot10^{-19}$
$^{237}Np \rightarrow {}^{233}Pa$	450	2607	3058	$2.08\cdot10^{-19}$
$^{233}U \rightarrow {}^{229}Th$	447	2676	3122	$2.12\cdot10^{-19}$
$^{229}Th \rightarrow {}^{225}Ra$	440	2752	3165	$2.15\cdot10^{-19}$
$^{225}Ac \rightarrow {}^{221}Fr$	452	3263	3714	$2.52\cdot10^{-19}$
$^{221}Fr \rightarrow {}^{217}At$	477	3565	4042	$2.74\cdot10^{-19}$
$^{217}At \rightarrow {}^{213}Bi$	483	3994	4477	$3.04\cdot10^{-19}$
$^{213}Bi \rightarrow {}^{209}Tl$[a]	466	3422	3888	$2.64\cdot10^{-19}$
$^{213}Po \rightarrow {}^{209}Pb$	510	4758	5268	$3.58\cdot10^{-19}$
**^{242}Pu decay chain**				
$^{242}Pu \rightarrow {}^{238}U$	443	2606	3049	$2.07\cdot10^{-19}$
**^{238}Pu decay chain**				
$^{238}Pu \rightarrow {}^{234}U$	414	2717	3131	$2.13\cdot10^{-19}$
$^{234}U \rightarrow {}^{230}Th$	420	2451	2871	$1.95\cdot10^{-19}$
$^{230}Th \rightarrow {}^{226}Ra$	419	2424	2843	$1.93\cdot10^{-19}$
$^{226}Ra \rightarrow {}^{222}Rn$	414	2514	2927	$1.99\cdot10^{-19}$
$^{222}Rn \rightarrow {}^{218}Po$	418	2893	3311	$2.25\cdot10^{-19}$
$^{218}Po \rightarrow {}^{214}Pb$	419	3212	3631	$2.46\cdot10^{-19}$
$^{214}Po \rightarrow {}^{210}Pb$	447	4069	4516	$3.07\cdot10^{-19}$
$^{210}Po \rightarrow {}^{206}Pb$	407	2962	3369	$2.29\cdot10^{-19}$
**^{239}Pu decay chain**				
$^{239}Pu \rightarrow {}^{235}U$	419	2559	2979	$2.02\cdot10^{-19}$
**^{240}Pu decay chain**				
$^{240}Pu \rightarrow {}^{236}U$	421	2551	2971	$2.02\cdot10^{-19}$
$^{236}U \rightarrow {}^{232}Th$	406	2303	2709	$1.84\cdot10^{-19}$

**^{241}Pu decay chain**				
$^{241}Am \rightarrow ^{237}Np$	417	2690	3107	$2.11 \cdot 10^{-19}$
$^{237}Np \rightarrow ^{233}Pa$	420	2420	2839	$1.93 \cdot 10^{-19}$
$^{233}U \rightarrow ^{229}Th$	417	2484	2900	$1.97 \cdot 10^{-19}$
$^{229}Th \rightarrow ^{225}Ra$	410	2528	2938	$1.99 \cdot 10^{-19}$
$^{225}Ac \rightarrow ^{221}Fr$	421	3027	3448	$2.34 \cdot 10^{-19}$
$^{221}Fr \rightarrow ^{217}At$	444	3309	3753	$2.55 \cdot 10^{-19}$
$^{217}At \rightarrow ^{213}Bi$	450	3706	4156	$2.82 \cdot 10^{-19}$
$^{213}Bi \rightarrow ^{209}Tl$ [a]	434	3176	3610	$2.45 \cdot 10^{-19}$
$^{213}Po \rightarrow ^{209}Pb$	475	4417	4892	$3.32 \cdot 10^{-19}$
**^{242}Pu decay chain**				
$^{242}Pu \rightarrow ^{238}U$	413	2418	2831	$1.92 \cdot 10^{-19}$

[a] branching decay: $^{213}Bi \rightarrow ^{209}Tl$ (2.2 %), $^{213}Bi \rightarrow ^{213}Po \rightarrow ^{209}Pb$ (97.8 %)

Table S3. Isotopic composition of Pu produced in nuclear reactors for different burn-up levels and reactor designs (HWR: Heavy water reactor; AGR: Advanced gas-cooled reactor, BWR: Boiling-water reactor, PWR: Pressurised-water reactor, VVER: Pressurised water-water energetic reactor, MOX: mixed oxide $(U,Pu)O_2$ fuel; n.a.: information not available—isotope produced but only in small amounts).

Reactor type	Burn-up / GWd t_{HM}^{-1}	^{238}Pu / wt%	^{239}Pu / wt%	^{240}Pu / wt%	^{241}Pu / wt%	^{242}Pu / wt%	Ref.
Magnox	3	0.1	80.0	16.9	2.7	0.3	[112]
Magnox	5	n.a.	68.5	25.0	5.3	1.2	[112]
CANDU	7.5	n.a.	66.5	26.5	5.3	1.5	[112]
HWR	10	0.1	60.7	29.9	6.9	2.4	[113]
AGR	18	0.6	53.7	30.8	9.9	5.0	[112]
BWR	22	1.0	57.3	20.6	15.9	5.2	[113]
BWR	27.5	2.6	59.8	23.7	10.6	3.3	[112]
BWR	30.4	n.a.	56.8	23.8	14.3	5.1	[112]
PWR	33	1.3	56.6	23.2	13.9	4.7	[112]
PWR	40	2.1	53.8	25.2	13.1	5.9	[113]
PWR	43	2.0	52.5	24.1	14.7	6.2	[112]
PWR	53	2.7	50.4	24.1	15.2	7.1	[112]
PWR	55	4.0	50.4	23.0	12.3	9.1	[114]
PWR	65	4.8	47.5	23.8	12.1	10.5	[114]
VVER-400	25	1.0	60.1	21.6	12.4	5.3	[113]
VVER-1000	33	1.4	56.4	22.8	14.1	5.3	[113]
PWR MOX	43	2.8	37.4	32.6	18.2	9.0	[115]

Table S4. Calculated (integral) alpha doses (D_{alpha}) and dose rates for waste forms containing 10 wt% Pu waste load originating from different sources (Pu isotopic composition from Table S3) compared to ^{239}Pu.

**Reactor type (Burn-up)**	**D_{alpha} in ... years / α g^{-1}**				**Dose rate during ... years / α g^{-1} s^{-1}**			
	**10^3 y**	**10^4 y**	**10^5 y**	**10^6 y**	**10^3 y**	**10^4 y**	**10^5 y**	**10^6 y**
Magnox (3 GWd t_{HM}^{-1})	$1.55 \cdot 10^{19}$	$8.50 \cdot 10^{19}$	$2.40 \cdot 10^{20}$	$2.66 \cdot 10^{20}$	$4.93 \cdot 10^{8}$	$2.70 \cdot 10^{8}$	$7.62 \cdot 10^{7}$	$8.45 \cdot 10^{6}$
Magnox (5 GWd t_{HM}^{-1})	$2.17 \cdot 10^{19}$	$9.73 \cdot 10^{19}$	$2.40 \cdot 10^{20}$	$2.75 \cdot 10^{20}$	$6.87 \cdot 10^{8}$	$3.09 \cdot 10^{8}$	$7.62 \cdot 10^{7}$	$8.71 \cdot 10^{6}$
CANDU (7.5 GWd t_{HM}^{-1})	$2.19 \cdot 10^{19}$	$9.88 \cdot 10^{19}$	$2.40 \cdot 10^{20}$	$2.75 \cdot 10^{20}$	$6.95 \cdot 10^{8}$	$3.13 \cdot 10^{8}$	$7.61 \cdot 10^{7}$	$8.71 \cdot 10^{6}$
HWR (10 GWd t_{HM}^{-1})	$2.58 \cdot 10^{19}$	$1.05 \cdot 10^{20}$	$2.39 \cdot 10^{20}$	$2.83 \cdot 10^{20}$	$8.19 \cdot 10^{8}$	$3.32 \cdot 10^{8}$	$7.59 \cdot 10^{7}$	$8.97 \cdot 10^{6}$
AGR (18 GWd t_{HM}^{-1})	$3.27 \cdot 10^{19}$	$1.11 \cdot 10^{20}$	$2.36 \cdot 10^{20}$	$3.01 \cdot 10^{20}$	$1.04 \cdot 10^{9}$	$3.51 \cdot 10^{8}$	$7.49 \cdot 10^{7}$	$9.55 \cdot 10^{6}$
BWR (22 GWd t_{HM}^{-1})	$4.33 \cdot 10^{19}$	$1.12 \cdot 10^{20}$	$2.37 \cdot 10^{20}$	$3.31 \cdot 10^{20}$	$1.37 \cdot 10^{9}$	$3.56 \cdot 10^{8}$	$7.51 \cdot 10^{7}$	$1.05 \cdot 10^{7}$
BWR (27.5 GWd t_{HM}^{-1})	$3.78 \cdot 10^{19}$	$1.10 \cdot 10^{20}$	$2.43 \cdot 10^{20}$	$3.37 \cdot 10^{20}$	$1.20 \cdot 10^{9}$	$3.48 \cdot 10^{8}$	$7.70 \cdot 10^{7}$	$1.07 \cdot 10^{7}$
BWR (30.4 GWd t_{HM}^{-1})	$3.83 \cdot 10^{19}$	$1.11 \cdot 10^{20}$	$2.35 \cdot 10^{20}$	$3.08 \cdot 10^{20}$	$1.22 \cdot 10^{9}$	$3.51 \cdot 10^{8}$	$7.45 \cdot 10^{7}$	$9.77 \cdot 10^{6}$
PWR (33 GWd t_{HM}^{-1})	$4.07 \cdot 10^{19}$	$1.12 \cdot 10^{20}$	$2.38 \cdot 10^{20}$	$3.27 \cdot 10^{20}$	$1.29 \cdot 10^{9}$	$3.55 \cdot 10^{8}$	$7.53 \cdot 10^{7}$	$1.04 \cdot 10^{7}$
PWR (40 GWd t_{HM}^{-1})	$4.13 \cdot 10^{19}$	$1.13 \cdot 10^{20}$	$2.38 \cdot 10^{20}$	$3.37 \cdot 10^{20}$	$1.31 \cdot 10^{9}$	$3.60 \cdot 10^{8}$	$7.53 \cdot 10^{7}$	$1.07 \cdot 10^{7}$
PWR (43 GWd t_{HM}^{-1})	$4.40 \cdot 10^{19}$	$1.15 \cdot 10^{20}$	$2.36 \cdot 10^{20}$	$3.41 \cdot 10^{20}$	$1.39 \cdot 10^{9}$	$3.64 \cdot 10^{8}$	$7.48 \cdot 10^{7}$	$1.08 \cdot 10^{7}$
PWR (53 GWd t_{HM}^{-1})	$4.66 \cdot 10^{19}$	$1.17 \cdot 10^{20}$	$2.36 \cdot 10^{20}$	$3.53 \cdot 10^{20}$	$1.48 \cdot 10^{9}$	$3.70 \cdot 10^{8}$	$7.48 \cdot 10^{7}$	$1.12 \cdot 10^{7}$
PWR (55 GWd t_{HM}^{-1})	$4.39 \cdot 10^{19}$	$1.11 \cdot 10^{20}$	$2.32 \cdot 10^{20}$	$3.61 \cdot 10^{20}$	$1.39 \cdot 10^{9}$	$3.52 \cdot 10^{8}$	$7.35 \cdot 10^{7}$	$1.14 \cdot 10^{7}$
PWR (65 GWd t_{HM}^{-1})	$4.55 \cdot 10^{19}$	$1.12 \cdot 10^{20}$	$2.31 \cdot 10^{20}$	$3.72 \cdot 10^{20}$	$1.44 \cdot 10^{9}$	$3.56 \cdot 10^{8}$	$7.31 \cdot 10^{7}$	$1.18 \cdot 10^{7}$
VVER-400 (25 GWd t_{HM}^{-1})	$3.66 \cdot 10^{19}$	$1.07 \cdot 10^{20}$	$2.36 \cdot 10^{20}$	$3.17 \cdot 10^{20}$	$1.16 \cdot 10^{9}$	$3.39 \cdot 10^{8}$	$7.50 \cdot 10^{7}$	$1.00 \cdot 10^{7}$
VVER-1000 (33 GWd t_{HM}^{-1})	$4.11 \cdot 10^{19}$	$1.12 \cdot 10^{20}$	$2.37 \cdot 10^{20}$	$3.29 \cdot 10^{20}$	$1.30 \cdot 10^{9}$	$3.55 \cdot 10^{8}$	$7.52 \cdot 10^{7}$	$1.04 \cdot 10^{7}$
PWR MOX (43 GWd t_{HM}^{-1})	$5.40 \cdot 10^{19}$	$1.30 \cdot 10^{20}$	$2.35 \cdot 10^{20}$	$3.68 \cdot 10^{20}$	$1.71 \cdot 10^{9}$	$4.13 \cdot 10^{8}$	$7.47 \cdot 10^{7}$	$1.17 \cdot 10^{7}$
pure ^{239}Pu	$7.13 \cdot 10^{18}$	$6.29 \cdot 10^{19}$	$2.38 \cdot 10^{20}$	$2.54 \cdot 10^{20}$	$2.26 \cdot 10^{8}$	$1.99 \cdot 10^{8}$	$7.54 \cdot 10^{7}$	$8.04 \cdot 10^{6}$

References for Supplementary Material

102. Brown, D.A.; Chadwick, M.B.; Capote, R.; Kahler, A.C.; Trkov, A.; Herman, M.W.; Sonzogni, A.A.; Danon, Y.; Carlson, A.D.; Dunn, M.; Smith, D.L.; Hale, G.M.; Arbanas, G.; Arcilla, R.; Bates, C.R.; Beck, B.; Becker, B.; Brown, F.; Casperson, R.J.; Conlin, J.; Cullen, D.E.; Descalle, M.-A.; Firestone, R.; Gaines, T.; Guber, K.H.; Hawari, A.I.; Holmes, J.; Johnson, T.D.; Kawano, T.; Kiedrowski, B.C.; Koning, A.J.; Kopecky, S.; Leal, L.; Lestone, J.P.; Lubitz, C.; Marquez Damian, J.I.; Mattoon, C.M.; McCutchan, E.A.; Mughabghab, S.; Navratil, P.; Neudecker, D.; Nobre, G.P.A.; Noguere, G.; Paris, M.; Pigni, M.T.; Plompen, A.J.; Pritychenko, B.; Pronyaev, V.G.; Roubtsov, D.; Rochman, D.; Romano, P.; Schillebeeckx, P.; Simakov, S.; Sin, M.; Sirakov, I.; Sleaford, B.; Sobes, V.; Soukhovitskii, E.S.; Stetcu, I.; Talou, P.; Thompson, I.; Marck, S. van der; Welser-Sherrill, L.; Wiarda, D.; White, M.; Wormald, J.L.; Wright, R.Q.; Zerkle, M.; Zerovnik, G.; Zhu, Y. ENDF/B-VIII.0: The 8th major release of the Nuclear Reaction Data Library with CIELO-project cross sections, new standards and thermal scattering data. Nucl. Data Sheets 2018, 148, 1–142.
112. OECD-NEA, Plutonium Fuel: An assessment; OECD Nuclear Energy Agency: Paris, France, 1989.
113. Wallenius, M.; Peerani, P.; Koch, L. Origin determination of plutonium material in nuclear forensics. J. Radioanal. Nucl. Chem. **2000**, *246*, 317-321.
114. OECD-NEA, Physics of plutonium recycling, vol. 1 – Issues and perspectives; OECD Nuclear Energy Agency: Paris, France, 1995.
115. Kang, J.; Hippel, F.N. von; MacFarlane, A.; Nelson, R. Storage MOX: A third way for plutonium disposal? Science and Global Security **2002**, *10*, 85-101.
